# Strong and Tough MXene-Induced Bacterial Cellulose Macrofibers for AIoT Textile Electronics

**DOI:** 10.1007/s40820-025-02046-y

**Published:** 2026-01-07

**Authors:** Yi Hao, Zixuan Zhang, Yajun Chen, Song Wang, Yingjia Tong, Pengfei Lv, Qufu Wei, Chengkuo Lee

**Affiliations:** 1 Key Laboratory of Special Protective Textiles, Ministry of Education, College of Textile Science and Engineering, Jiangnan University, Wuxi, 214122 People’s Republic of China; 2 Department of Electrical and Computer Engineering, National University of Singapore, Singapore, 117583 Singapore; 3 Center for Intelligent Sensors and MEMS (CISM), National University of Singapore, Singapore, 117583 Singapore; 4 Suzhou Institute of Trade & Commerce, Suzhou, 215009 People’s Republic of China; 5 State Key Laboratory for Mechanical Manufacturing Systems Engineering, Xi’an Jiaotong University, Xi’an, 710049 People’s Republic of China; 6 School of Life Sciences and Health Engineering, Jiangnan University, Wuxi, 214122 People’s Republic of China; 7 Laboratory of Flexible Electronics Technology, Tsinghua University, Beijing, 10084 People’s Republic of China

**Keywords:** Textile electronics, MXene/PEODT:PSS ink, Bacterial cellulose macrofiber, Triboelectric nanogenerators, Liquid recognition

## Abstract

Textile electronics with extraordinary sensing capabilities holds significant potential in the Artificial Intelligence of Things (AIoT). However, little effort is paid to their mutual advantages of robust interfacial interactions, ultra-strong mechanical performance, and stability. Herein, we fabricate homogeneous and multifunctional core–shell macrofibers by integrating bridge-functionalized MXene/PEDOT:PSS conductive ink with aligned bacterial cellulose (BC). These resulting macrofibers feature mechanical properties (tensile strength of 433.2 MPa and the Young’s modulus of 25.9 GPa), exceptional electrical conductivity (10.05 S cm^−1^) and durable hydrophobicity. Such superior robustness allows for the fabrication of the macrofibers woven into textile-based triboelectric nanogenerator (PKT-TENG) and shows an impressive high-performance of a maximum open-circuit voltage of 272.54 V, short-circuit current of 14.56 μA and power density of 86.29 mW m^−2^, which successfully powers commercial electronics. As the proof-of-concept illustration, the macrofibers with durable hydrophobicity and high piezoresistive sensitivity are further employed for precepting diverse liquids that can simultaneously monitor their distinctive motion features via real-time resistance variation on the textile-based array. This work is expected to offer new insights into the design of advanced fibers with ultra-strong mechanical capabilities and high conductivity and provide an avenue for the development of textile electronics for high-performance sensing and intelligent manufacturing.

**Supplementary Information:**

The online version contains supplementary material available at 10.1007/s40820-025-02046-y.

## Highlights

- PKT-TENG woven with K-MXene/PEDOT:PSS integrated bacterial cellulose (BC) via polydimethylsiloxane (PDMS) coating (PKMPBC) macrofibers were fabricated by bridging K-MXene/PEDOT:PSS ink with aligned BC macrofibers, then dip-coated with PDMS, showing high conductivity (10.05 S cm^−1^), high mechanical strength (433.8 MPa) and superior Young’s modules (25.9 GPa).
- PKT-TENG integrated with PKMPBC macrofiebrs shows excellent triboelectric response and stability, delivering 86.29 mW m^−2^ power density to power an electronic watch and capacitors.
- Resistance-sensitive PKMPBC macrofibers proved the capability of recognition for diverse liquid with precisely detection and fed back multifactor behaviors.

**Supplementary Information:**

The online version contains supplementary material available at 10.1007/s40820-025-02046-y.

## Graphical abstract

**Figure:**
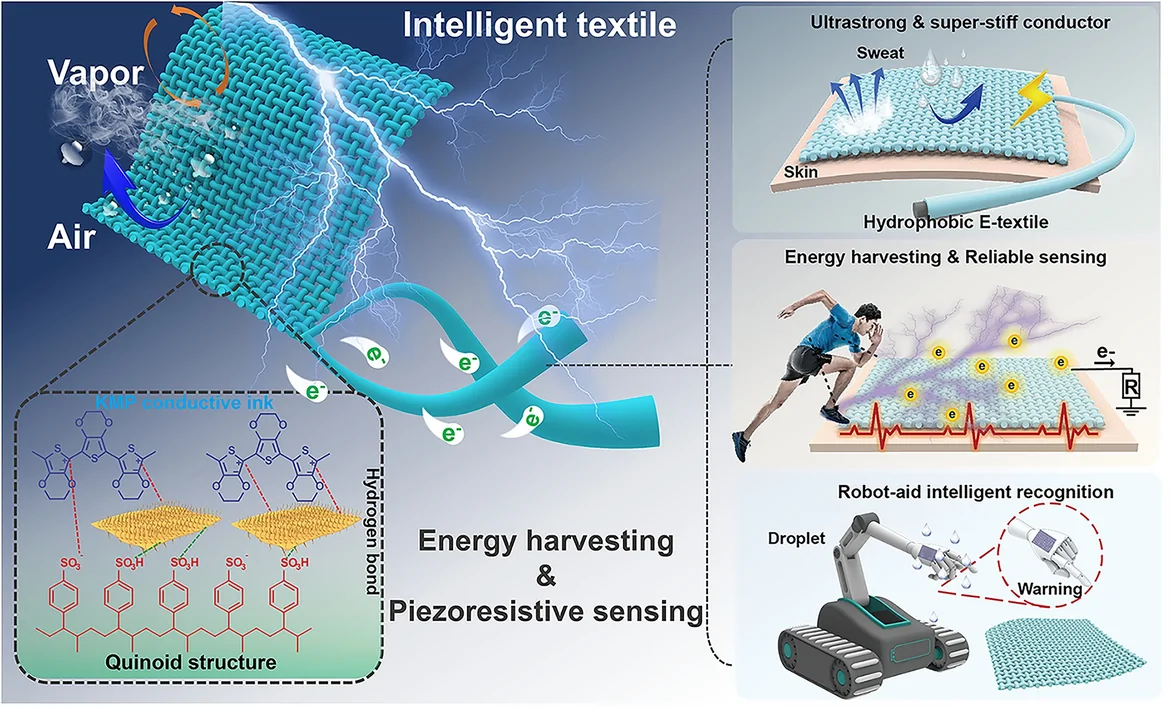

## Introduction

Electronic textiles, which are made from fibers ranging from traditional textile fibers to advanced intelligent fibers, have emerged as a next-generation technology with promising applications in bio-integrated devices that demand mechanical adaptability, portability, lightweight, washability and comfort [1–3]. In particular, flexible, deformable and easily manufacturable electronic textiles have attracted significant attention due to the demand for comfort in practical use [4–7]. Moreover, advanced intelligent fiber materials and their integrated electronics have demonstrated remarkable advantages in military, reconnaissance, emergency rescue, exploration of unknown environments and assistive devices due to their unique perception capabilities. These advanced fiber-based systems provide the potential to deliver timely, accurate and reliable information and essential support for the formulation and implementation of strategic plans. A wide range of textile electronics based on piezoelectric [8], triboelectric [9], capacitive [10] and resistive [11] sensing mechanisms have been extensively developed to achieve sensory capabilities comparable to or even surpassing those of human skin [12–14]. These flexible textile electronics or fiber-based sensing arrays are capable of detecting a variety of external physical stimuli, such as mechanical pressure [15], strain [16], temperature [17], ambient humidity [18] and even motion frequency [19]. However, the above-mentioned devices are typically limited to solid or air media due to their weak hydrophobicity, which restricts their ability to reliably perceive stimuli in liquid environment [20]. Indeed, liquids represent one of the most frequently encountered elements in the operational environment of wearable electronics. On the one hand, with the popularity of wearable electronics, a potential challenge is that textile electronics are susceptible to liquids (e.g., bodily fluids) that attack in the working environment, which may cause great trouble for device stability and maintenance, as well as the reliability of sensing signals [21]. On the other hand, intelligent robotic systems demand comprehensive access to environmental information, including gases, liquids, temperature, and airflow [22–26]. Among these, one of the critical factors is the detection of falling liquid, such as rain, dew or hazardous substances. It is of great significance to develop robust textile electronics with comprehensively perceive capabilities for mechanical energy harvesting and real-time environmental monitoring in wearables.

In recent advances, numerous electronic skins have been regarded as one of the most commonly used approaches for mimicking human perception, enabling the detection and discrimination of liquid samples. For example, a study proposed a polytetrafluoroethylene (PTFE) film-based bionic electronic tongue to achieve highly reliable and intelligent liquid identification by integrating triboelectric sensing and deep learning [27]. Researchers developed a dual-mode liquid sensor featuring a liquid-solid interface with an electric double-layer structure, which integrated piezoresistive and triboelectric sensing mechanisms for the identification of ionic solutions [28]. However, most of the flexible electronic skins reported to date for liquid monitoring are constructed with dense polymer matrix, which hinder the exchange of substances between human skin and the external environment, as well as the wearing comfort. Limited efforts have been devoted to develop advanced fiber materials with multifunctionality for liquid-recognition textile electronics. Moreover, mechanical robustness is a highly critical characteristic for high performance of textile electronics to ensure that the multifunctional system maintains a stable structure and functionality under mechanical deformation in practical use [29–32]. To achieve highly robust fiber materials, tough polymers or polymer composites are typically employed and processed through spinning followed by post-drawing techniques to endow the fibers with flexibility, stretchability and damage resistance [33, 34]. However, these strong fibers often possess limited functionality (e.g., poor electrical conductivity) and complex multi-step processes due to traditional polymer matrix, which restricts their applications in electronic textiles [35, 36]. Additionally, although synthetic conductive composite fibers have been proven to have excellent functionality by introducing functional inorganic or nanomaterials (such as graphene, Ti_3_C_2_T_x_ MXene, carbon nanotubes and conductive polymers) [37–41], it should be noted that the nanoconductive fillers within these fibers tend to aggregate and precipitate in soft material systems, especially when negatively charged MXene nanosheets are introduced [42, 43]. Moreover, the weak interlayer interactions and disordered structure limit the mechanical properties, conductivity and overall stability of MXene-based soft system [44–46].

Herein, we report a judicious strategy and synthesis of hydrophobic conductive and ultra-strong core-shell macrofiber electrode by bridging surface-functionalized MXene/PEDOT: PSS conductive ink and aligned bacterial cellulose (BC) macrofibers, followed by dip-coating with polydimethylsiloxane (PDMS). The MXene nanosheets are surface-functionalized with γ-methacryloxypro pyltrimethoxysilane (KH570) to form a mechanically interlocked structure with PEDOT: PSS inside the K-MXene/PEDEOT:PSS (KMP) conductive ink. The formation of homogeneous highly conductive macrofibers with strong interface interactions could be attributed to the strong π–π stacking interaction between K-MXene nanosheets and PEDOT: PSS, the transition of PEDOT chains from benzene phase to quinone phase as well as the hydrogen bonding interaction between KMP conductive ink and BC matrix. Especially, stretching-twisting and PDMS coating process was introduced to produce oriented macrofibers, with a degree of orientation 0.56. The advantages mentioned above endow the resulting macrofibers with three superiorities: 1) superior electrical conductivity (10.05 S cm^−1^ in air and 7.80 S cm^−1^ in deionized water); 2) outstanding load-bearing capacity (a strength of 433.8 MPa and high Young’s modulus up to 25.9 GPa); and 3) remarkable hydrophobicity with a contact angle of 142° and exceptional capable of biodegradation within 144 h. Furthermore, the PKT-TENG woven with K-MXene/PEDOT:PSS integrated BC via PDMS coating (PKMPBC) macrofibers inherits durable hydrophobicity and flexibility, and it possesses capacity of excellent triboelectric outputs of a maximum open-circuit voltage of 272.54 V, a short-circuit current of 14.56 μA and peak power density of 86.29 mW m^−2^. It successfully powered devices such as electronic watch and commercial capacitor and furtherly constructed an intelligent clothing and heath monitoring system to monitor human motions (e.g., walking, running, jumping, arm lifting and leg lifting). As a proof-of-concept illustration, the PKMPBC macrofibers with resistance sensitivity have proved to be capable of recognition for diverse liquid, which can precisely detect and feed back the multifactor behaviors of liquid droplets, and are expected to achieve the high-precision sensing and monitoring system in unmanned factories for liquid hazardous materials. This strategy can not only provide new insights into the fabrication of advanced fiber materials with high conductivity and self-powered sending capabilities, but also could open up a new avenue toward the development of textile electronics, intelligent soft robotics and intelligent factory, and beyond.

## Experimental Section

### Materials

Ti_3_AlC_2_ MAX powder with an average particle size of 400 meshes was purchased from Jilin 11 Technology Co., Ltd. Lithium fluoride (LiF, 99%), sodium acetate (≥ 99.0%), acetic acid (≥ 99.5%) sodium hydroxide (NaOH, ≥ 96.0%), hydrochloric acid (HCl, 36.0% ~ 38.0%), N, N-dimethylformamide (DMF, ≥ 99.5%), acetone (≥ 99.5%), ethyl alcohol (≥ 99.5%) and n-hexane were purchased from Sinopharm Chemical Reagent Co., Ltd (Shanghai, China). Bacterial cellulose hydrogel membranes were sourced from Guilin Qihong Technology Co., Ltd. Cellulase was obtained from Beijing Soleibao Technology Co., Ltd. γ-Methacryloxypropyltrimethoxysilane (KH570, 98%) was obtained from Beijing Innochem Co., Ltd. Poly(3,4-ethylenedioxythiophene)/poly(styrenesulfonate) (PEDOT:PSS, 1.5% in water, viscosity ≤ 350) was purchased from Shanghai Sain Chemical Technology Co., Ltd. Deionized (DI) water was used in all experiments. All the reagents were of analytical grade and used as received without further purification.

### Synthesis of Ti_3_C_2_T_x_ MXene and KH570-Modified MXene (K-MXene) Nanosheets

The delaminated Ti_3_C_2_T_x_ MXene nanosheets were synthesized by selectively etching the Al layer from MAX (Ti_3_AlC_2_) powder using a mixed acid method with LiF/HCl based on a modified minimum interaction layer delamination (MILD) method. As a result, the final sediments were redispersed in DI water, resulting in a 10 mg mL^−1^ concentration solution. The as-prepared MXene nanosheets were ultrasonically dispersed in 500 mL of DI water to form a homogeneous suspension. Subsequently, 1 mL of γ-methacryloxypropyltrimethoxysilane (KH570) was added dropwise at a controlled rate of 0.1 mL min^−1^ under continuous magnetic stirring (80 rpm) at room temperature. The reaction was maintained for 48 h to allow sufficient hydrolysis and condensation of KH570, resulting in the successful preparation of surface-functionalized KH570-modified MXene (K-MXene) nanosheets via silane coupling. The product was collected by centrifugation and washed three times with DI water at 8000 rpm for 5 min each to remove any unbound silane molecules.

### Synthesis of K-MXene/PEDOT: PSS Conductive Ink

K-MXene/PEDOT: PSS conductive ink was synthesized by adding a specific amount of PEDOT: PSS aqueous dispersion (1.5 wt%) into 20 mL of homogeneous K-MXene dispersion (10 mg mL^−1^) under continuous vigorous stirring in an ice water bath for 5 h. After a brief reaction, the dispersion in water was obtained and named as KM_x_P_y,_ in which the mass ratios of K-MXene to PEDOT: PSS were 3:7, 1:1, 7:3 and 1:0, respectively).

### Preparation of BC, KMPBC and PKMPBC Macrofibers

The BC hydrogel membranes were cut into strips measuring 20 cm × 0.6 cm and treated with boiling 0.1 wt% NaOH solution and DI water to remove impurities and fully expand the fiber network. The swollen BC hydrogel strips were then processed into long BC fibers through a stretching-twisting and drying process at room temperature. Next, BC hydrogel stripes were immersed in K-MXene/PEDOT: PSS conductive ink with different mass ratios, and K-MXene/PEDOT: PSS@BC (KMPBC) macrofibers were formed using the same stretching-twisting and drying process at room temperature. Then, the mixture (1.76 g) of uncross-linked PDMS matrix and cross-linked agent (with a mass ratio of 10:1) was dissolved in n-hexane (10 g) to prepare a 15 wt% dispersion. Finally, KMPBC macrofibers were immersed in the hydrophobic solution for 20 min and dried at room temperature (20 ± 3 °C, 40% ± 2%) to obtain hydrophobic PDMS/K-MXene/PEDOT: PSS@BC (PKMPBC) macrofibers.

### Fabrication of PKMPBC-based TENG (PKT-TENG) and Self-Powered Sensors

PKMPBC fibers were woven into a 1/1 plain woven fabric (10 cm × 7 cm) using traditional Chinese sewing techniques to serve as a single-electrode PKT-TENG, which was used individually and integrated into a pair of smart pants. Additionally, various fabrics and materials, including PTFE, PVDF, PDMS, PET, PVC, nylon and cotton, were used alternative triboelectric materials to prepare PKT-TENG in a dual-electrode mode, in combination with the PKMPBC fabric. The liquid prediction and recognition sensing array were assembled by adhering PKMPBC macrofibers onto VHB adhesive tape (4905, 3 M). The real-time signal transmission and sensing system were controlled by external devices, with the electrical response signals measured using an electrometer (Keithley 6514) and controlled by a custom LabVIEW program. The real-time resistance responded signals were recorded and collected on a data acquisition board in Python on VS code platform.

### Degradation of BC Macrofibers and PKMPBC Macrofibers

An acetic acid–sodium acetate buffer with a pH of 4.8 was prepared by uniformly mixing 150 mL of 0.2 M sodium acetate mother liquor with 100 mL of 0.2 M acetic acid mother liquor and then diluting to 1 L with DI water. To prepare the cellulase solution, 0.5 g of cellulase was dissolved in 100 mL of the above-prepared HAc-NaAc buffer solution. Then, BC macrofibers and PKMPBC macrofibers were, respectively, soaked in the cellulase solution and degraded in a 50 °C water bath. Optical photographs were taken every 12 h from the start of the degradation experiment until the macrofibers were completely degraded to compare their morphology during the degradation process. Additionally, the available large macrofibers were collected to observe their morphology with SEM and calculate the weight loss during the degradation process.

### Characterizations and Measurement

High-resolution transmission electron microscopy (HR-TEM) images of MXene and K-MXene nanosheets were obtained on an FEI Talos F200X G2 at an accelerating voltage of 200 kV. The surface morphology and microstructure of monolayer MXene and K-MXene nanosheets were observed using atomic force microscopy (AFM, Dimension Icon, Bruker) under a beam acceleration of 200 kV. The microstructure and corresponding energy-dispersive *X*-ray spectroscopy (EDX) elemental mapping of the samples were collected using a field emission scanning electron microscope (FESEM, SU8100) at an acceleration voltage of 10 kV. The chemical structures were observed by *X*-ray photoelectron spectroscopy (XPS, K-Alpha, Thermo Scientific). The rheological properties of the ink at 25 °C were recorded on a rotational rheometer (Anton Paar MCR301, parallel plate geometry, 50 mm diameter, 0.5 mm gap). The change of viscosity with the shear rate range from 0.01 to 100 s^−1^ was measured by using the logarithmic step method. The composition and crystal structure of the synthesized sample were recorded by *X*-ray diffraction (XRD, AXS D2 PHASER, Bruker) with a scanning range from 5° to 70°. Dynamic light scattering (DLS) was performed using a Malvern Zetasizer Nano series (Zetasizer Nano ZS) to determine the hydrodynamic particle size. The thermogravimetric analysis curve (TGA) was collected on the thermogravimetric analyzer (TGA, METTLER) with a heating rate of 20 °C min^−1^ and a temperature range from 50 to 800 °C within a high-purity nitrogen atmosphere. The static water contact angle (WCA) was measured using the sessile drop method on a contact angle measuring instrument (DCAT-21, Dataphysics) with a 10-μL droplet. The mechanical tensile performance of macrofiber samples was evaluated by an electronic universal material testing machine (5967X, INSTRON) with a tensile speed of 10 mm min^−1^. Wide-angle X-ray scattering (WAXS) and small-angle *X*-ray scattering (SAXS) of samples were obtained on a Xeuss 2.0 instrument (Xenocs, CuKα radiation, *λ* = 1.54 Å at 50 kV and 60 mA, beam perpendicular to sample, exposure time 180 s). The cyclic contact-separation process was carried out using a linear motor (Linmot C1100), and the corresponding output performance of the textile-based TENG was evaluated by an electrometer (Keithley 6514). The charging and discharging experiments of commercial capacitors were conducted on an electrochemical workstation (CHI 660E). The conductivity of the composite macrofibers was tested using a linear sweep voltammetry (LSV) and is calculated via Eq. 1:

1 $$\sigma = \frac{L}{{{\mathrm{RS}}}}$$

where σ is the electrical conductivity; *L*, R, and S represent the length, resistance, and cross-sectional area of the macrofiber, respectively. The weaving of PKMPBC fiber was achieved through the traditional Chinese sewing technique. At the same time, the corresponding movies were recorded by a Canon EOS 90D DSLR camera. The bioelectrical signals were measured under the temperature of 20 ± 3 °C and the humidity of 40% RH.

## Results and Discussion

### Synthesis and Characterizations of K-MXene/PEDOT:PSS-Integrated BC Macrofibers

**Fig. 1:**
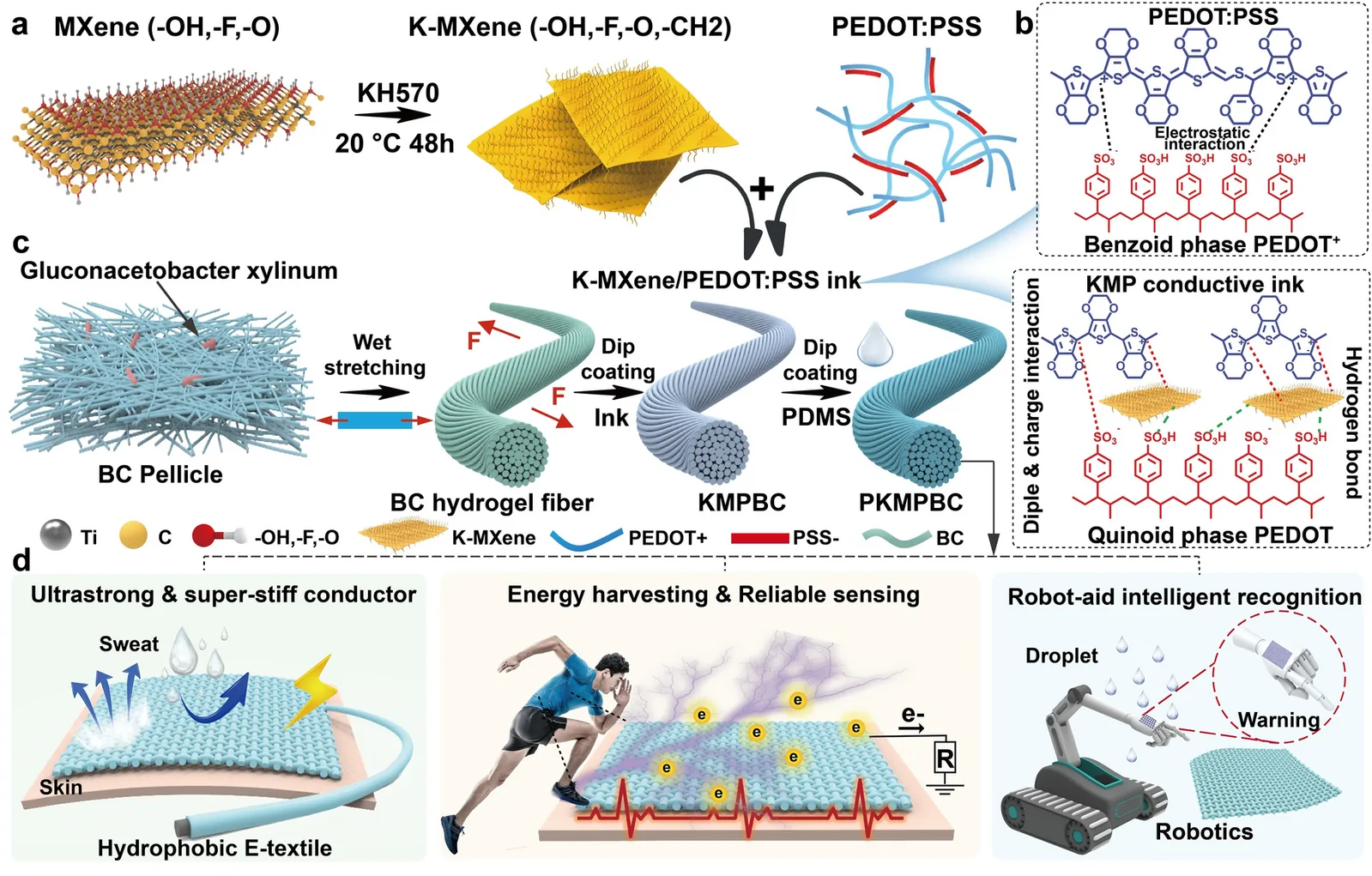
Synthesis and fabrication of K-MXene/PEDOT:PSS-integrated BC macrofibers.** a** Fabrication schematic of the K-MXene/PEDOT:PSS conductive ink. **b** Schematic diagram of electron transfer and structural change from benzene to quinone of PEDOT:PSS. **c** Fabrication process of the ultra-strong and conductive PKMPBC macrofibers through layer-by-layer approach with continuously assembled by dip-coating. **d** PKMPBC macrofibers with superb property and diverse promising applications in ultra-strong and super-stiff conductor, energy harvesting and reliable sensing, as well as robot-aid intelligent recognition of liquids, respectively

The homogeneous macrofiber system with markable electrical conductivity is fabricated by in situ copolymerization of K-MXene/PEDOT:PSS conductive ink with BC hydrogel macrofibers after stretching-twisting and drying processes (Fig. S1). First, Ti_3_C_2_T_x_ MXene nanosheets are synthesized by selectively etching Al layer in Ti_3_AlC_2_ MAX with the modified minimally intensive layer delamination (MILD) method [47, 48]. KH570-MXene (K-MXene) is then developed by surface-functionalized MXene with KH570 in aqueous solution (Figs. 1a and S2-S8). The hydrolysis reaction converts Si-OCH_3_ groups of KH570 into Si-O groups, resulting in the hydrogen bonds between the silanol bond in KH570 and -OH surface terminal group of MXene. The condensation reaction accompanied by the formation of Si-O covalent bonds occurs between MXene and KH570. Second, the resulting K-MXene nanosheets are rapidly mixed with the PEDOT:PSS aqueous dispersion to obtain the highly stable K-MXene/PEDOT:PSS conductive ink with appropriate viscosity and rheological properties, in which the positively charged PEDOT chain segments electrostatic interacted with negatively charged K-MXene (Fig. 1b). Subsequently, the stripe BC hydrogel is immersed into K-MXene/PEDOT:PSS conductive ink via electrostatic self-assembly in the procedure, followed by a stretching-twisting process and further drying to obtain the K-MXene/PEDOT:PSS-integrated BC macrofibers (KMPBC). Final super-strong and highly electroconductive PKMPBC macrofibers are successfully fabricated by encapsulating KMPBC macrofibers within a uniform PDMS shell, which not only provides robust mechanical reinforcement but also ensures environmental stability and functional durability (Fig. 1c). After four steps above, the integration of PKMPBC macrofibers yields a super-strong textile electronics and further it was used for self-powered sensing and monitoring systems. The representative proof-of-concept demonstrates motion posture detection, micro-nano-energy harvesting, hazardous liquid monitoring and warning, human motion monitoring systems and biodegradable textile electronics, as shown in Fig. 1d.

**Fig. 2:**
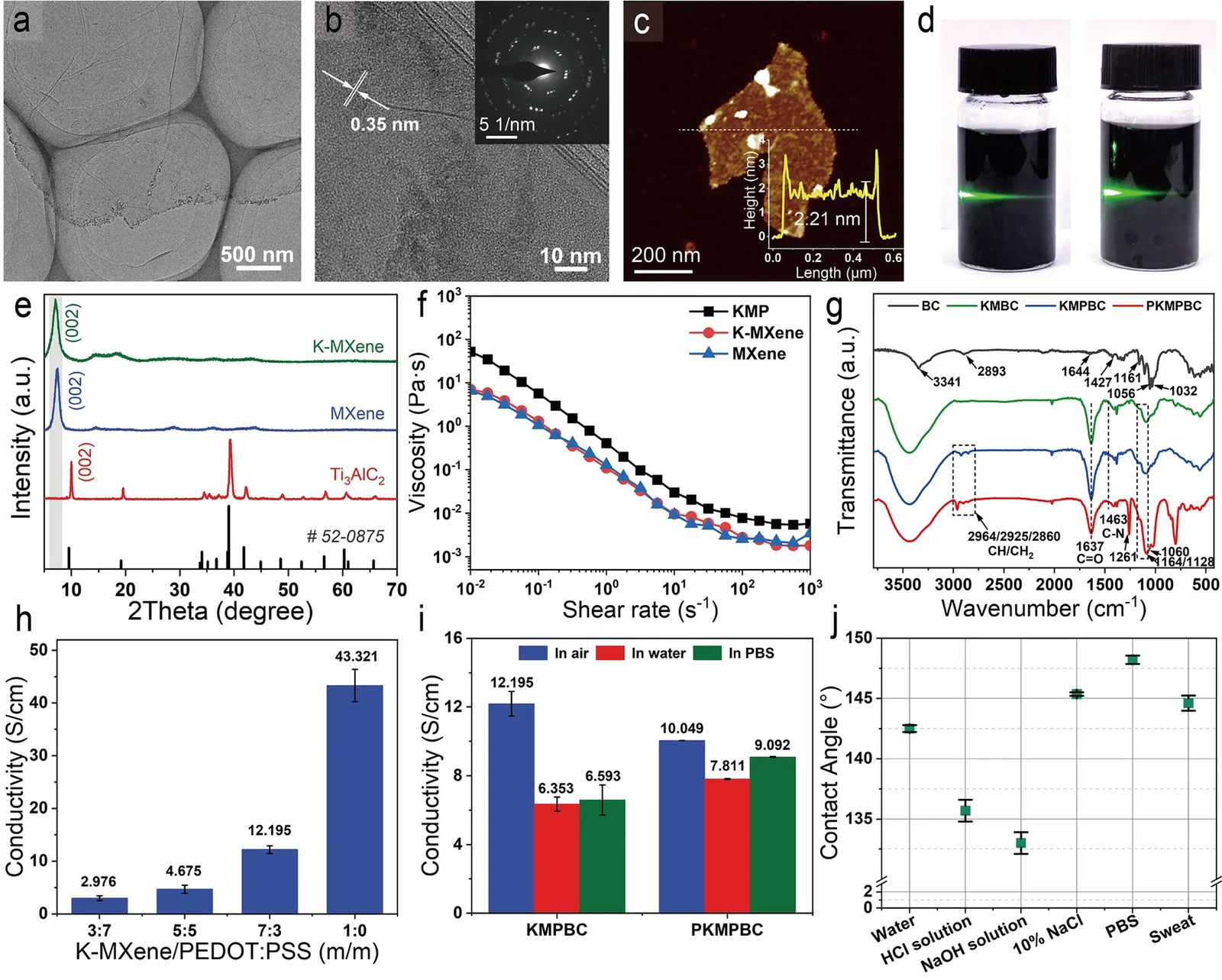
Characterization and properties of K-MXene, KMP ink and PKMPBC macrofibers. **a, b** High-resolution transmission electron microscopy (HR-TEM) images, the corresponding SAED pattern and (101) fringe spacing of K-MXene nanosheet, **c** AFM image of K-MXene. **d** MXene and K-MXene nanosheets dispersion with a distinct Tyndall scattering effect. **e** XRD pattern of MXene before (blue) and after (green) surface-functionalized with KH570. **f** KMP conductive ink viscosity versus shear rate. **g** FTIR spectra of BC, KMBC, KM_7_P_3_BC and PKM_7_P_3_BC macrofibers. **h** Conductivity comparison of KM_x_P_y_BC macrofibers with different mass ratios of K-MXene and PEDOT:PSS. **i** Electrical conductivity of KM_7_P_3_BC macrofiber and PKM_7_P_3_BC macrofiber. **j** The contact angle values of PKM_7_P_3_BC macrofibers to water, solutions of HCl, NaOH, NaCl, PBS and artificial sweat group, respectively

Transmission electron microscopy (TEM) confirms the successful etching and delamination of Ti_3_C_2_T_x_ MXene nanosheets (Figs. S2 and S3). And the surface-functionalized nanosheets of K-MXene show an extremely thin and almost transparent microstructure in Figs. 2a-b and S6. Lattice fringes with an interplanar spacing of 0.35 nm are observed in the high-resolution transmission electron microscopy (HR-TEM) image of K-MXene nanosheets, which corresponds to the (101) plane of K-MXene [49]. As illustrated in Fig. 2c, the height profile obtained from the atomic force microscope (AFM) image reveals that the thickness of the K-MXene nanosheets is approximately 2.21 nm. A distinct Tyndall effect can be observed in the dilute dispersion of K-MXene nanosheets in Fig. 2d, demonstrating the excellent hydrophilicity and dispersibility of K-MXene nanosheets. The exfoliated Ti_3_C_2_T_x_ MXene nanosheets exhibits a thickness of approximately 1.42 nm (Fig. S3), which is larger than the theoretical thickness of single-layer MXene because of the adsorption of water molecules on the MXene surface. XRD patterns confirm the successful synthesis of Ti_3_C_2_T_x_ MXene nanosheets and the crystal structure changes of K-MXene as illustrated in Fig. 2e. The characteristic diffraction peak (002) of Ti_3_C_2_T_x_ MXene shifts to a lower angle (2θ = 8.05°) compared with the MAX phase (2θ = 9.99°). The absence of the (104) peak of Ti_3_AlC_2_ MAX phase at around 39° in the *X*-ray diffraction curve of Ti_3_C_2_T_x_ MXene proves the complete etching of the aluminum atomic layer in Ti_3_AlC_2_ MAX [50]. In addition, the characteristic diffraction peak of (002) further shifts from 8.05° for Ti_3_C_2_T_x_ MXene to 7.07° for K-MXene. Compared to Ti_3_C_2_T_x_ MXene, K-MXene shows an even greater interlayer spacing of 1.25 nm, indicating that KH570 modification leads to an expansion of the interlayer distance. Raman spectra are performed to analyze the molecular-level interactions among PEDOT:PSS, K-MXene/PEDOT:PSS conductive ink and PKMPBC macrofibers as shown in Fig. S9. Pristine PEDOT:PSS exhibits a prominent peak at 1430 cm^−1^, corresponding to the symmetric stretching vibration of *C*_*α*_ = *C*_*β*_ on the pentathiophene ring of PEDOT, an ethylenedioxy bridge characteristic band of PEDOT at ≈983 cm^−1^, and the PSS characteristic band at ≈436 cm^−1^ [51]. Notably, a significant red shift (≈8 cm^−1^) of the 1430 cm^−1^ peak is observed in both K-MXene/PEDOT:PSS conductive ink and the PKMPBC macrofibers because of a structural transition of the PEDOT segment from a benzenoid to a quinoid configuration. This could be interpreted as a relief of physical restriction of the oxyethylene ring in PEDOT upon cross-linking with K-MXene nanosheets, enabling more free molecular vibrations [52]. The transition from a coiled benzenoid phase to linear/extended-coil quinoid phase in the ground state may lead to a higher delocalization on PEDOT:PSS, thereby achieving a higher charge carrier density [53]. Ultimately, the incorporation of K-MXene nanosheets into PEDOT:PSS induces a phase transition of the PEDOT segments from the benzenoid to the quinoid phase due to strong π-stacking interactions between negativity charged K-MXene and positivity charged PEDOT. Dynamic light scattering characterization confirms that the average hydrodynamic diameters of PEDOT:PSS are about 345.79 nm, while the size of PEDOT:PSS in KMP conductive ink is reduced (Fig. S10a, b). Furthermore, KMP conductive ink exhibits a higher zeta potential compare to PEDOT:PSS dispersion (Fig. S10c), indicating excellent stability of the conductive KMP conductive ink. As a result, the issues of aggregation and precipitation in the system are significantly improved, and a high conductivity is achieved due to the increased quinone structure of PEODT. However, K-MXene dispersion exhibits poor rheological performance due to the weak interlayer interactions and the small lateral size of the nanosheets. Rheological behaviors of K-MXene and K-MXene/PEDOT:PSS ink are evaluated as shown in Fig. 2f. Conductive inks of MXene, K-MXene and K-MXene/PEDOT:PSS exhibit both shear thinning and non-Newtonian fluid properties, and viscosity decreases with an increasing shear rate. At the same low MXene concentration (10 mg mL^−1^), the viscosities of MXene, K-MXene and KMP ink are 6.55, 6.98, and 51.65 Pa s, respectively. The maximum viscosity of K-MXene/PEDOT:PSS ink is an order of magnitude higher than that of the original MXene dispersion, which is mainly attributed not only to the formation of dipoles and electrostatic interactions between the abundant surface terminal groups of the K-MXene nanosheets and the polar groups of PEDOT:PSS, but also because of the limitation of the relative slip of nanosheets when PEDOT:PSS is inserted into ordered arrangement nanosheet layers. To reveal the interaction between K-MXene/PEDOT:PSS and BC macrofibers, the Fourier-transform infrared (FTIR) spectra of K-MXene, PEDOT:PSS and PKMPBC macrofibers are obtained in Figs. 2g and S11. The peak at 3425 cm^−1^ is attributed to the vibration of the -OH group on the K-MXene surface, while the absorption peak at 1718 cm^−1^ corresponds to the C=O bond of KH570. Three consecutive broad peaks between 1000 and 1200 cm^−1^ are attributed to the presence of -Si-O- [54]. Bacterial cellulose is well known for the FTIR peaks at 3341 and 1644 cm^−1^, corresponding to the -OH stretching and bending modes, respectively [55]. In addition, FTIR spectra show a red shift of the -OH stretching vibration from 3341 cm^−1^ in BC to 3270 cm^−1^ in PKMPBC, indicating the formation of hydrogen bonds between the hydroxyl groups of BC and the sulfonic acid groups of PSS. The intensified absorption peaks at 1164/1128 cm^−1^ and 1032 cm^−1^ corresponding to the S=O stretching of PSS indicate the formation of hydrogen bonds between the sulfonic acid groups of PSS and K-MXene, as well as PSS and BC molecular chains. Besides, the thermostabilities of MXene and macrofibers are further evaluated. The thermal weight loss ratio of PKM_7_P_3_BC macrofibers is 46.8%, as shown in Fig. S12.

Furthermore, the electrical conductivity of PKMPBC macrofibers is one of the crucial factors that determines both output charge amount and sensing signal sensitivity, as higher conductivity facilitates charge transfer. The electrical conductivity of PKMPBC macrofiber is calculated by Eq. 1. With the increase of K-MXene content, the conductivity of PKMPBC macrofiber improves from 2.98 S cm^−1^ of KM_3_P_7_BC to 4.67 S cm^−1^ of KM_5_P_5_BC, 12.20 S cm^−1^ of KM_7_P_3_BC and 43.32 S cm^−1^ of KMBC macrofibers (Fig. 2h). To further test the conductivity of KM_7_P_3_BC macrofibers and PKM_7_P_3_BC macrofibers, the resistance and conductivity of the samples are measured and calculated in air, water and PBS buffer, respectively (Fig. 2i). The electrical conductivity of the PKMPBC macrofibers under high-temperature/high-humidity conditions and after immersion in a dilute HCl acid solution was evaluated. As indicated in Fig. S13, the electrical conductivity reached 9.04 S cm^−1^ at 40 ± 2 °C, 90% ± 3% and 7.86 S cm^−1^ after immersion in a dilute HCl solution. PBS solution is used to simulate the environment of bodily fluids such as sweat. For the in-air group, the conductivity of KMPBC macrofibers is 12.20 S cm^−1^, while that of PKM_7_P_3_BC macrofibers exhibits a lower value of 10.05 S cm^−1^, which can be attributed to the protection formed by the PDMS shell structure between the macrofibers and surrounding air. The conductivity of KM_7_P_3_BC macrofibers in water and PBS buffer is significantly lower than that in air, as well as than that of PKM_7_P_3_BC macrofibers in the same situations, suggesting that water and PBS have a pronounced impact on the electrical conductivity of KM_7_P_3_BC macrofibers. However, this phenomenon in conductivity is not observed in PKM_7_P_3_BC macrofibers; liquid cannot penetrate into the interior of PKM_7_P_3_BC macrofibers owing to the protection provided by the hydrophobicity layer. Therefore, the electrical conductivity of PKM_7_P_3_BC macrofibers is more reliable and stable in liquid environment than that of KM_7_P_3_BC macrofibers. The contact angle measurements are conducted in water, 10% HCl solution, 10% NaOH solution, 10% NaCl solution, PBS and artificial sweat to simulate exposure to hazardous liquids, including acidic, alkaline and saline environments. Figure 2j depicts the variation in contact angles of PKM_7_P_3_BC macrofibers with the aforementioned liquids. The corresponding contact angles are 142.5° (water), 127.4° (HCl), 132.7° (NaOH), 145.3° (NaCl), 148.2° (PBS) and 144.5° (artificial sweat), respectively. The measured contact angle images are presented in Fig. S14.

### Mechanical Properties of the PKM_7_P_3_BC Macrofibers

**Fig. 3:**
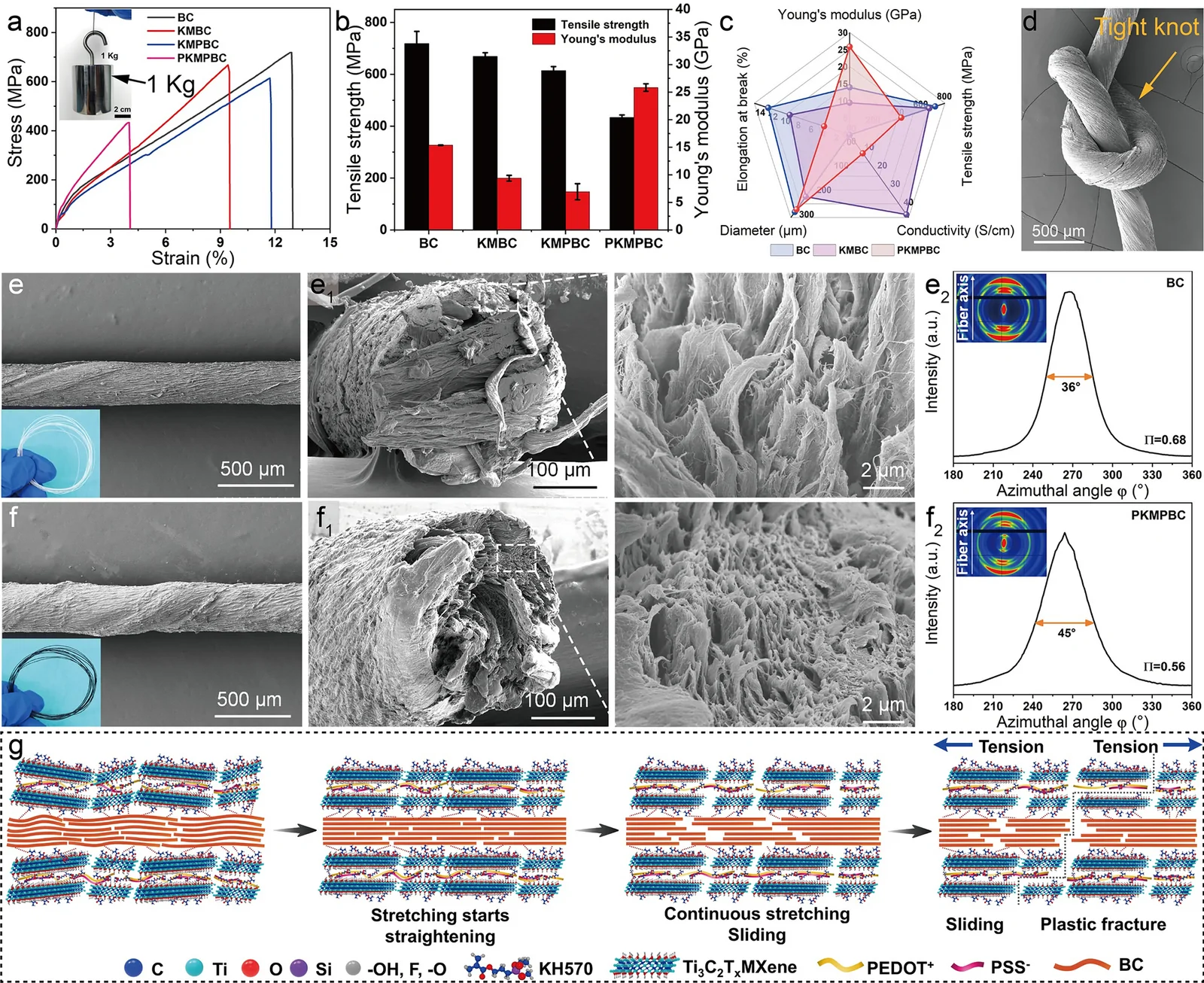
Mechanical properties and fracture mechanism of PKMPBC macrofibers. **a** Typical stress-strain curves, **b** tensile strength and Young’s modulus of BC, KMBC, KMPBC and PKM_7_P_3_BC macrofibers.** c** Comparison of mechanical properties and between the PKM_7_P_3_BC macrofibers and other MXene materials. **d** SEM image of a knotted PKM_7_P_3_BC fiber. **e–f** SEM images and photographs of pure BC and PKM_7_P_3_BC macrofibers. **e**_**1**_**-f**_**1**_ Cross-sectional SEM images of pure BC and PKM_7_P_3_BC macrofibers. **e**_**2**_**-f**_**2**_ 2D WAXS images with corresponding *fwhm* curves for the BC and PKM_7_P_3_BC macrofibers. **g** Proposed fracture process of PKM_7_P_3_BC macrofibers

Given the rigorous weaving processes and application requirements of textile-based sensing systems, an outstanding mechanical robustness is essential to accommodate bending, impact and abrasion. To validate the superior performance of the composite macrofibers, Fig. 3a, b investigates the typical stress-strain curves and the corresponding tensile strength, the Young’s modulus of the BC, KMBC, KM_7_P_3_BC and PKM_7_P_3_BC macrofibers. Thanks to the highly aligned approach using stretching-twisting processes, the fabricated BC macrofibers provide the highest tensile strength compared with other composite macrofibers. The corresponding tensile strength of 718.4 MPa and the Young’s modulus of 15.4 GPa, which is much higher than that of undrawn BC macrofibers (a tensile strength of 122.0 MPa and a Young’s modulus of 5.8 GPa), are shown in Fig. S15. The loose structure of cellulose composite nanofibers of KMBC and KM_7_P_3_BC has the decreased tensile strength of 668.6 and 610.4 MPa and the Young’s modulus of 9.4 and 6.9 GPa, respectively. The alignment of nanosheets, densification of sheet and conductive polymers, and less bound water in PKM_7_P_3_BC macrofibers ensure greater water-cellulose hydrogen bonds, resulting in a tendency to assemble with each other into more compact microfiber bundles leading to a more compact structure with a superb Young’s modulus of 25.9 GPa and a tensile strength of 433.8 MPa, which is still superior to previous conductive macrofibers (Fig. 3c and Table S1). As displayed in Fig. S16, the mechanical strength tests of PKMPBC macrofibers under high-temperature/high-humidity conditions and after immersion in HCl solution demonstrate the mechanical stability under extreme environments, exhibiting tensile strength of 478.8 and 301.4 MPa, respectively. More intuitively, the exceptional mechanical strength and durability features enable a single PKM_7_P_3_BC macrofibers lift a weight of 1 kg, which is more than 30,000 times of than the macrofiber (Fig. S17). Additionally, PKM_7_P_3_BC macrofibers can be easily folded, even tightly knotted without fracture as illustrated in Fig. 3d.

Furthermore, the as-fabricated macrofibers with perfect integrity and well-alignment structure can be observed from the cross-sectional SEM images of resulting samples (Figs. 3e_1_-f_1_ and S18a_1_-a_2_). The cross-sectional morphology of the PKM_7_P_3_BC macrofibers shows a tightly bound and well-aligned structure, which is similar to that of the BC macrofibers, KMBC macrofibers, KM_7_P_3_BC macrofibers as well as PKM_7_P_3_BC macrofibers. The fracture morphologies of the BC, KMBC, KM_7_P_3_BC and PKM_7_P_3_BC macrofibers are shown in enlarged images in Figs. 3e_1_-f_1_, S18a_1_-a_2_. For the BC macrofibers, the large BC nanofibril bundles are pulled out and exhibit a typical axially elongated structure. It is worth noting that the sufficient amount of free water between macrofibers is crucial for the rearrangement of cellulose molecular chains during wet-drawing, serving as lubricant between nanofibers and allowing for the movement of cellulose molecular chains. In contrast, the PKM_7_P_3_BC macrofibers show a close-packed aligned morphology with a small degree of nanofibril bundle elongation owing to the stretching-twisting combined with solvent displacement by *n*-hexane in PDMS dispersion, which can significantly reduce the hydration state by extruding free water from the macrofibrous hydrogel with hydrogel state. Clearly, nanofibers tend to gather tightly together and assemble into interlocked microfiber bundles through hydrogen bonding between fibers with the wet-stretching, wet-twisting approach and water evaporation as shown in the cross-sectional morphology. Wide-range *X*-ray (WAXS) and small-angle *X*-ray scattering (SAXS) measurements (Figs. 3e_2_, f_2_, S18a_2_, b_2_ and S19) are further performed to elucidate the microscopic structure of macrofibers via inducing *X*-rays perpendicular to the fiber axis. The Herman orientation factor (ƒ) is derived from the azimuthal intensity distribution corresponding to the (002) diffraction peak obtained in the WAXS measurements [56, 57], which increases from 0.53 for KMBC macrofibers to the 0.56 for PKM_7_P_3_BC macrofibers (Fig. S19). Also, PKM_7_P_3_BC macrofibers possess the smallest half-maximum (*fwhm*) of 45°, validating the largest orientation alignment among the as-prepared composite macrofibers. The proposed fracture mechanism of PKM_7_P_3_BC macrofiber is proposed in Fig. 2g. During the initial stretching, the tensile force straightens the slightly curved BC microfibrils after stretching-twisting process and unordered K-MXene nanosheets and PEDOT:PSS polymer chains, and the alignment and orientation degree are robustly enhanced with the tensile strain that continues loading. The tough BC core layer is largely elongated to the maximum extent by stretching the microfibers. Reasonably, a significant interlayer sliding occurs in K-MXene nanosheets to adapt to large deformations, and the tightly wrapped PDMS shell provides a restraining force that enables stable interlayer sliding of K-MXene nanosheets rather than fracture. The strong interaction between K-MXene nanosheets, PEDOT:PSS polymer chains and BC macrofiber core facilitates effective load transfer and dissipates significant energy for the fiber fracture. As stretching progresses, initial cracking occurs in the PKM_7_P_3_BC macrofiber owing to the sliding neighboring BC microfibrils, resulting in the failure of the hydrogen bonding. This is followed by the breakdown of the BC and K-MXene/PEDOT:PSS layers until the entire model fractures. Therefore, the high sheet orientation, dense stacking, strong interlayer interactions as well as ordered orientation of BC microfibers are responsible for the extraordinary mechanical properties of PKM_7_P_3_BC macrofibers.

### Construction and Output Performance of TENG Based on PKM_7_P_3_BC Integrated Textile

**Fig. 4:**
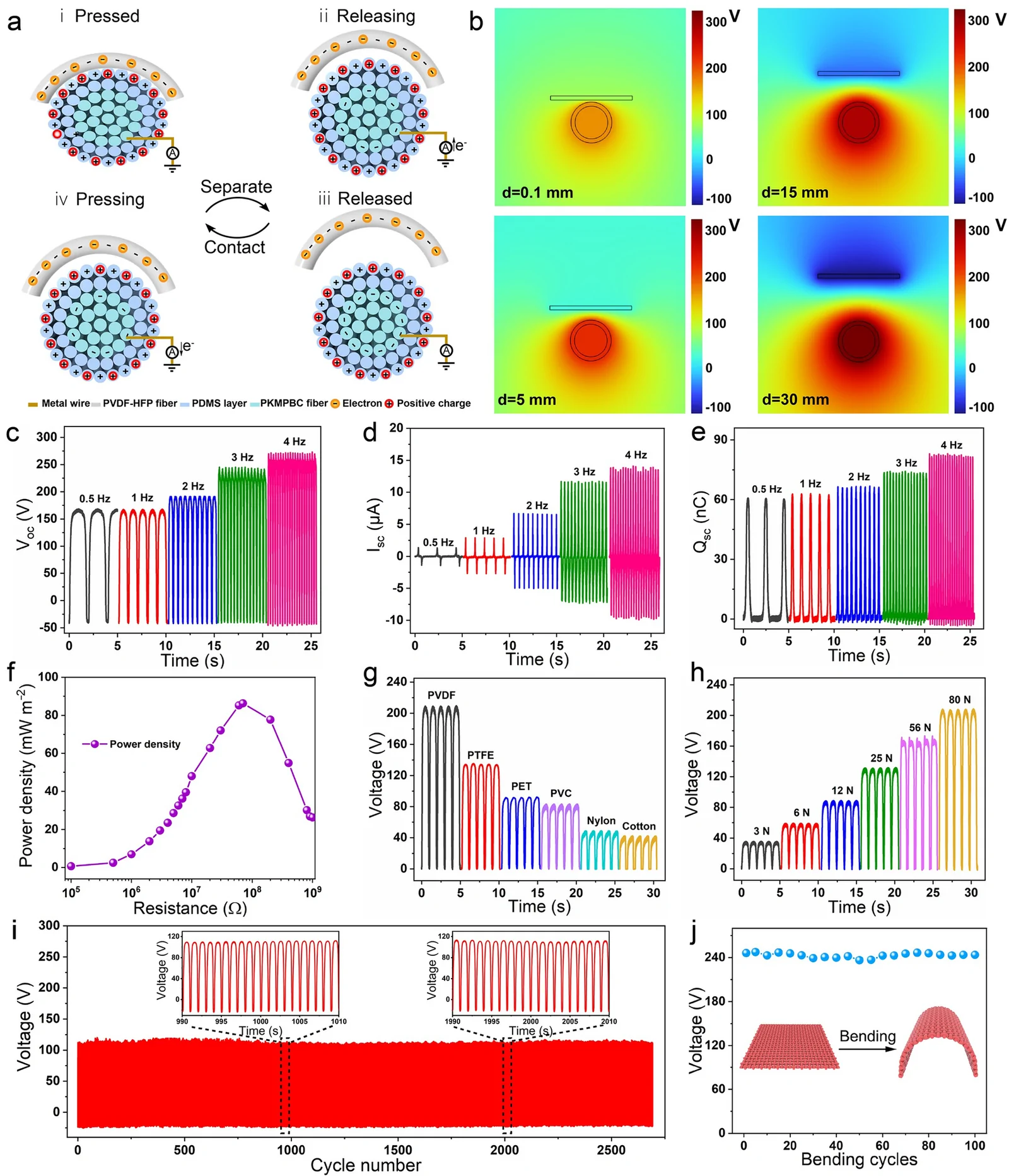
**a** Schematic diagram of the working principle of PKT-TENG, **b** numerical calculation of potential distribution of PKT-TENG with a contact-separation distance of 30 mm,** c** open-circuit voltage, **d** short-circuit current and **e** transferred charge of PKT-TENG in various operation frequencies. **f** Instantaneous power density related to external load resistance. **g** Output voltage of PKT-TENG paired with PVDF-HFP, PTFE, PET, PVC, nylon and cotton. **h** Instantaneous power density of PKT-TENG under different applied forces. **i** Long-term testing of PKT-TENG over 2700 contact–separation cycles at the frequency of 1 Hz. **j** Output voltage of PKT-TENG under 100 cycles of mechanical deformation

To assess the feasibility of the robust and multifunctional PKM_7_P_3_BC macrofibers in practical scenarios, it is first employed as a durable electromechanical sensing device for wearable electronics to monitor and distinguish stable biomechanical signals from human body. The designed textile-based TENG operates in two different modes based on the coupling effects of contact electrification and electrostatic induction: (1) contact-separation mode and (2) single-electrode mode. Materials with opposite electronegativity are selected to construct a textile-based TENG for optimal output performance. Electrospun PVDF-HFP nanofibers are employed as a representative highly electronegative material, as the rich C-F polar bonds and electrospinning-induced *β*-phase crystallization endow them with pronounced surface electronegativity and strong electron-accepting capability (Fig. S20). In the contact-separation mode, PKM_7_P_3_BC and PVDF-HFP electrospun nanofibers served as the triboelectric layers, while PKM_7_P_3_BC macrofibers and copper foil attached to the PVDF-HFP nanofiber membrane are used as electrodes. Base on the difference in electron affinity and electronegativity, electrons transfer from PDMS shell on the surface of PKM_7_P_3_BC with weaker electronegativity to PVDF-HFP nanofibers with strong electronegativity upon contact, forming opposite charges at the interface. During separation, the formed interfacial difference induces charge flow between electrodes through the external circuit, enabling energy conversion (Fig. S21a). When the applied external force is released, the tribopositive and tribonegative layers are separated, resulting in a potential difference between them and causing electrons flowing from copper electrode to the PKM_7_P_3_BC textile to balance the potential difference. Electrostatic equilibrium is established and electron flow stops when the two triboelectric layers are completely separated. Subsequently, electrons flow back from the ground to balance the potential difference once more when the PDMS shell of PKM_7_P_3_BC comes into contact with the PVDF-HFP nanofibrous layer again. Therefore, the PKT-TENG generates alternating current (AC) signals through the continuous contact-separation process. In the single-electrode mode, the standalone PKM_7_P_3_BC textile serves both as the triboelectric layer and the electrode to form a textile-based TENG, which could combine with the triboelectric layer (such as PVDF-HFP) that tends to obtain electrons to generate electrical sensing signals (Figs. 4a and S21b). Electrons flow from the PKM_7_P_3_BC textile along copper wires to the ground, as there is no conductive connection between the PKM_7_P_3_BC textile and the PVDF-HFP nanofibrous layer to forming a circuit. Then, electrons flow back from the ground through copper wire to the PKM_7_P_3_BC textile to equalize the potential difference when the PKM_7_P_3_BC textile comes close to the PVDF-HFP nanofibrous membrane again. Hence, single-electrode mode of PKT-TENG is more suitable as a human motion sensor. In the subsequent experiments, the triboelectric performance is tested in the contact-separation mode, on the basis of which a single-electrode mode was designed for human motion sensors in smart garments.

The electrical output of PKT-TENG is evaluated under cyclic contact-separation by a linear motor, with device dimensions of 7 cm × 10 cm and a contact-separation spacing of 30 mm. Figure 4b shows the potential distribution simulated by COMSOL Multiphysics, quantitatively illustrating the power generation process during the contact-separation process of triboelectric charging. During the contact-separation process, the potential difference rises to a maximum of ~ 295.0 V as the separation distance increases from 0.1 to 30 mm. The open-circuit voltage (*V*_oc_), short-circuit current (*I*_sc_) and charge transfer quantity (*Q*_sc_) of the PKT-TENG are investigated at different contact-separation frequencies ranging from 0.5 to 4.0 Hz as shown in Fig. 4c, d. As the contact–separation frequency increases from 0.5 to 4.0 Hz, V_oc_ amplitude rises from 168.1 to 272.5 V, the *Q*_sc_ increases from 60.7 to 83 nC and *I*_sc_ grows from 1.3 to 14.6 μA. The output voltage testing photograph of PKT-TENG is shown in Fig. S22. Generally, the output power density of TENG depends on the external resistance. Figure S23 shows the variations in output voltage and current related to the load resistance as the load resistance ranges from 0.1 to 1000 MΩ. The output voltage of the PKT-TENG increases with the increase in load resistance, while the output current decreases as the load resistance increases. Figure 4f shows the instantaneous power density calculated via Eq. 2:

2 $$W = \frac{P}{A} = \frac{{U^{2} }}{{{\mathrm{RA}}}}$$

where *W* is the instantaneous power density, *P* is the output power, *R* is the value of load resistance,* U* is the output voltage and A is the effective contact area, respectively. Notably, the output power density initially exhibits an increasing trend, reaching a maximum instantaneous power density of 86.3 mW m^−2^ at a load resistance of 70 MΩ and then exhibits a declining trend. To clarify the impact of different triboelectric materials paired with PKM_7_P_3_BC textile on the output performance of PKT-TENG, various materials including PVDF-HFP, PTFE, PET, PVC, nylon and cotton are assembled into TENG to measure the corresponding output voltage (Fig. 4g). All test groups exhibited a measured voltage exceeding 40 V. Considering the maximum output voltage and the unique advantages of textile-based advanced materials in the wearable devices, PVDF-HFP nanofibrous membrane demonstrates an ideal candidate as a triboelectric material of textile-based TENGs in a dual-electrode mode. Furthermore, it may encounter external forces and complex mechanical deformations as a wearable textile-based TNEG. Therefore, it is essential to evaluate the impact of applied forces and deformations on its output performance. Figure 4h depicts the real-time electrical signals of *V*_oc_ under different applied forces. As the applied force increases from 3 to 80 N, *V*_oc_ steadily increases from 35 to 208 V. This is attributed to the increased deformation of the PKT-TENG under greater external force, which leads to an enhanced contact area between the triboelectric materials. In addition, long-term operational stability is an essential indicator for evaluating the performance of textile-based TENGs. As shown in Fig. 4i, no noticeable attenuation in the voltage signal is observed after more than 2700 contact-separation cycles and 100 large-scale twisting and stretching cycles as shown in Fig. 4j. As shown in Fig. S24, PKMPBC macrofibers exhibit no significant structural collapse or damage before and after 2700 contact-separation cycles, demonstrating their outstanding stability for practical applications. Figure S25 shows the *V*_oc_ output of the PKT-TENG before and after liquid pouring. The minimal change in electrical output after liquid pouring further demonstrates its excellent stability in practical applications.

### PKT-TENG as Self-Powered Sensor for Human Motion Monitoring

**Fig. 5:**
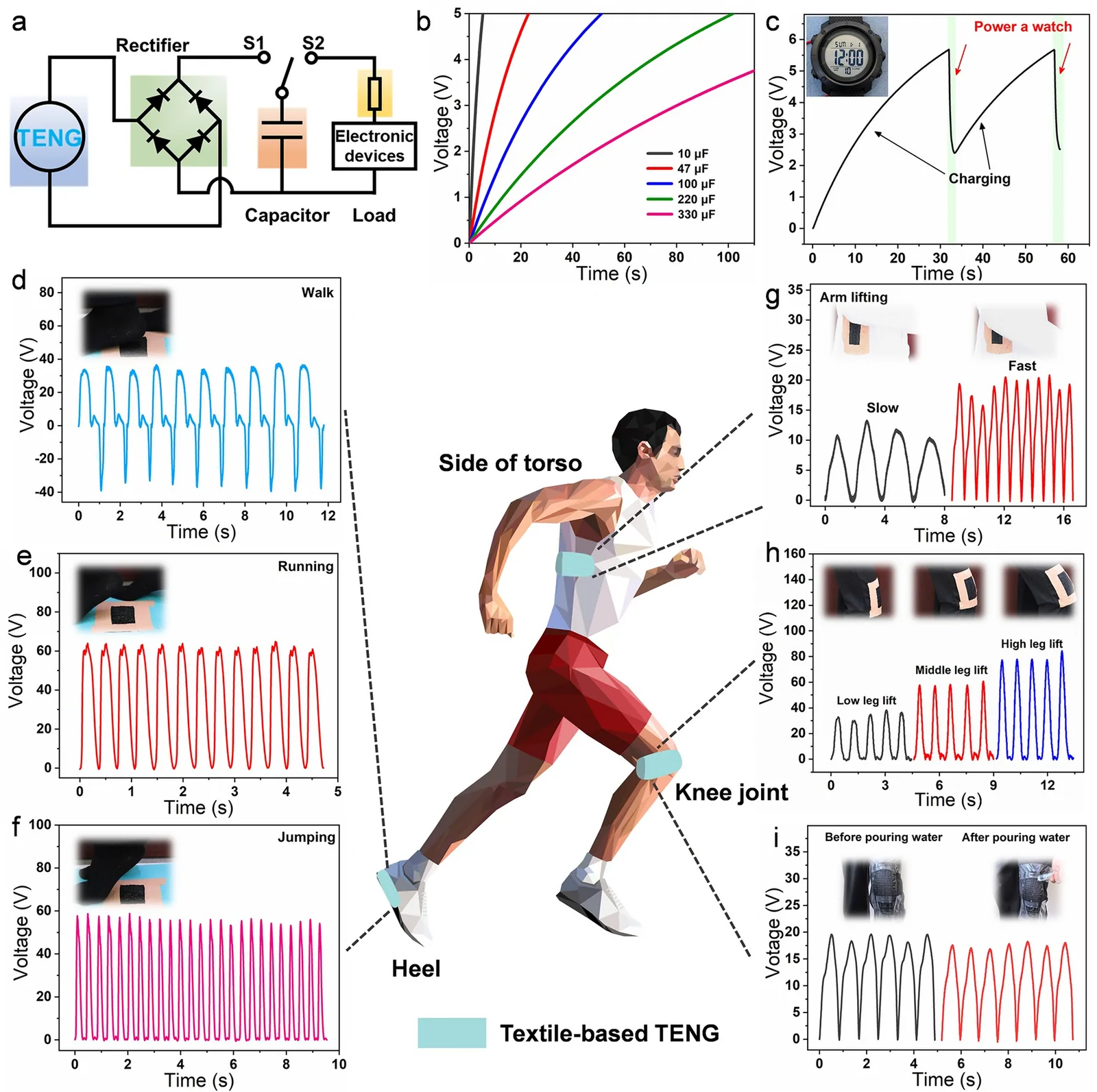
Performance of intelligent clothing and motion monitoring system. **a** Schematic diagram of the equivalent circuit for the PKT-TENG charging capacitors and powering electronic devices. **b** Charging curves of commercial capacitors (10, 47, 100, 220 and 330 μF) by operating PKT-TENG. **c** Real-time testing of capacitor voltage and powering a watch. The photographs and output voltages of PKT-TENG are tested as a self-powered sensor fixed to various parts of the human body (heel, side of the torso and other sensitive joints) to monitor real-time motion signals of mechanical movements: **d** walking, **e** running, **f** jumping, **g** arm lifting at different speeds, **h** leg lifting at different heights and **i** movements signals during liquid pouring conditions

PKT-TNEG shows multifunctional potentials as a power source for wearable and portable electronic devices and self-powered sensing based on its excellent mechanical properties and relatively high output performance (Movie S1). Typically, the electricity generated by the PKT-TENG can be stored in commercial capacitors with the output alternating current (AC) converted into direct current (DC) through a commercial rectifier bridge, as illustrated in the equivalent circuit diagram in Fig. 5a. Figure 5b shows the charging process of different capacitors (10, 47, 100, 200, and 300 μF) at a contact-separation frequency of 3 Hz. It is proved that the PKT-TENG can be used to continuously charge capacitors, while the charging rate decreases with the increase of capacitor capacity. Moreover, a 100 μF capacitor can be charged within 50 s under an operating frequency of 0.5 to 3 Hz in Fig. S26. The capacitor can be charged to 5.7 V within 32 s during continuous contact-separation motion and subsequently power an electronic watch (Fig. 5c). All these results indicate that PKT-TENG has significant application potential for driving electronic devices without external energy supply, particularly in the field of wearable electronics.

PKT-TENG has relatively lower electrical output in single-electrode mode but is strongly correlated with mechanical displacement. Therefore, we further fabricated sensing textile based on PKM_7_P_3_BC textile that can accurately monitor the type and intensity of human motion, making it ideal as a wearable self-powered sensor to monitor human motion. PKM_7_P_3_BC textile sensing system serves as an intelligent insole to identify the type of movement. For example, three different types of motions, walking, running and jumping, were used in the actual testing. A peak positive voltage of 37.5 V is generated at the maximum displacement of contact–separation process between the heel and the PKM_7_P_3_BC textile when walking. The maximum output *V*_*oc*_ reaches approximately 64.7 and 58.8 V during running and jumping, respectively, with the motion signal amplitude significantly increasing compared to waking. This is attributed to the increased collision force caused by higher running velocity, which significantly enhances the motion signals. Meanwhile, the two triboelectric layers cannot completely separate from each other, leading to a transition in the working mode of the single-electrode PKT-TENG from the contact–separation mode to a lateral sliding mode. Additionally, a gradual increase in the output voltage signal is observed, ranging from 13.2 to 20.8 V, as the arm lifting velocity changes from slow to fast. This evolution is caused by frequency variation between the clothing and PKM_7_P_3_BC textile fixed on to the torso, indicating a clear correlation between the signal amplitude and the arm lifting velocity (Fig. 5g). Figure 5h shows a positive correlation between leg lifting height and the electrical output signal, which may be due to a more complete contact-separation process as the leg lifting height increases. Figure 5i displays the electricity changes in knee joint motion signal before and after liquid pouring, with peak values of 19.4 and 18.2 V, demonstrating excellent hydrophobicity and operational stability. Furthermore, Movies S2, S3 demonstrate the detection of motion signals under the above conditions, identifying various motion states, as well as the stability of the output signal under liquid pouring.

### Augmented Perception of Liquid Classification by Intelligent Textile Systems

**Fig. 6:**
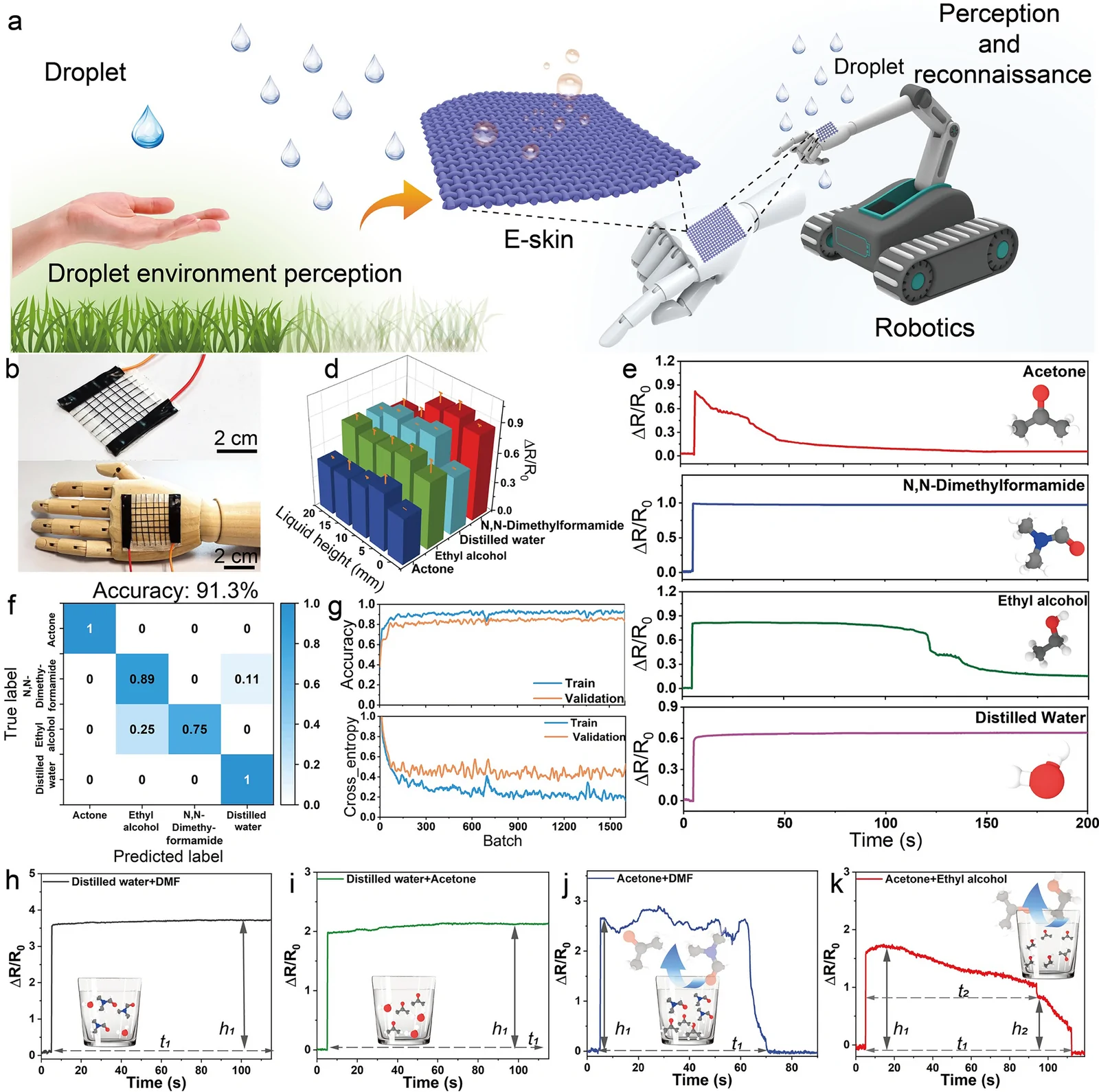
Depth perception and recognition feedback of multiple liquids. **a** Schematic diagram of biomimetic unmanned factory detection robotics inspired by human skin for detecting various liquids. **b** Photograph of the textile-based intelligent recognition sensory system of hazardous liquids. **c** Photograph of the intelligent textile recognition sensory system attached to a wooden prosthetic hand. **d** Resistance changes in the recognition system at different dropping heights of liquids applied.** e** Diverse resistance responses when different liquids, such as acetone, N, N-Dimethylformamide, ethyl alcohol and distilled water, are dropped onto a robotic hand covered with the recognition and perception system.** f** Confusion matrix from an actual single-attempt experiment and **g** the training history of the signal classification model. Variation in resistance responses under different mixed liquid systems: **h** distilled water and DMF, **i** distilled water and acetone, **j** acetone and DMF and **k** acetone and ethyl alcohol, respectively. *h*_*1*_, total relative resistance changes of mixed liquid; *h*_*2*_, relative resistance changes of ethyl alcohol; *t*_*1*_, total time required for volatilization; *t*_*2*_, time required for acetone volatilization

One of the ultimate goals of in-depth monitoring of complex liquid types and trajectories is to provide critical information for replacing human judgment in identifying hazards and taking acting in unmanned factories. In this regard, we purposefully designed and conducted experiments and developed a PKM_7_P_3_BC textile-based sensor with reliable monitoring capability and stale sensing response, enabling full-range monitoring of environmental stimuli to distinguish the dynamic behavior of liquids under various changing conditions. The PKM_7_P_3_BC textile-based sensor exhibits an outstanding sensing performance, including rapid response/recovery times of 45/68 ms and repeatable cyclic relative resistance changes under different loads ranging from 0.5 to 3 N (Fig. S27). The PKT textile exhibits excellent overall performance, and a combination properties comparison with other similar materials is summarized in Table S2. Figure 6a illustrates an intelligent detection robotic that can deeply perceive the type, volume and falling height of liquids and send alert to the operator. When an unknown liquid impacts the upper surface of the all PKM_7_P_3_BC textile array (3 cm × 2.5 cm), the sensing network exhibits detectable resistance variations due to time-dependent pressure fluctuations induced by the synergistic effects of gravitational acceleration and droplet evaporation dynamics (Figs. 6b and S28). The PKM_7_P_3_BC sensing system based on piezoresistive sensing mechanism is installed on a wooden prosthetic hand for identifying unknown liquids (Fig. 6c). Four liquids with typical differences in evaporation rates are used for practical testing. Among them, distilled water has low volatility due to the strong hydrogen bonding, resulting in a long residence time of the droplets on the sensor surface and a relatively stable resistance response. In contrast, high volatile organic liquids such as ethyl alcohol and acetone show rapid evaporation due to the lower intermolecular binding energy, leading to a short-lived but dynamic resistance decay process. Therefore, liquids exhibit unique resistance-time decay profile, which serves as the fundamental basis for classification in liquid-recognition system. Based on this, an intelligent liquid sensing and recognition system is established.

The intrinsic parameters and motion state of the liquids are systematically monitored under room temperature of 20 ± 3 °C and humidity of 40% ± 2%. For example, the volume of the liquids and the drop height are important parameters that must be monitored for e-skin on robotics. As illustrated in Fig. S29, the relative resistance change demonstrates a nonlinear behavior characterized by an initial increase followed by a gradual decline as the liquid volume expands from 10 to 100 μL. The relative resistance change is predominantly attributed to the deformation of the e-skin textile structure caused by liquid impact, and the maximum relative resistance response occurs at the maximum deformation. When PKM_7_P_3_BC textile become saturated with deformation, further increase in liquid volume will not change the resistance path, and the relative resistance will no longer increase. To ensure stable data acquisition for deep-learning-based liquid recognition, the droplet falling height is optimized prior to model training. Standardization and preprocessing procedures are subsequently applied to further suppress height-related variations, enabling the model to focus on the intrinsic characteristics of the liquids. As shown in Fig. 6d, the relative resistance changes of e-skin textile for liquids are explored at different heights (0, 5, 10, 15 and 20 mm, respectively). As the liquids falling height increases from 0 to 5 mm, the change of relative resistance increases significantly. In this case, the relative changes of Δ*R*/*R*_0_ for acetone, ethyl alcohol, distilled water and N, N-dimethylformamide (DMF) are 0.58, 0.76, 0.86 and 0.93, respectively as illustrated in Fig. 6e. These results indicate that liquids could achieve higher velocities at a certain height, thereby enhancing the resistance response performance. This capability can be extended to any type of liquid involved, including highly volatile organic liquids, such as acetone and ethyl alcohol, as well as less volatile liquid such as DMF and inorganic liquid such as distilled water. Herein, a neural network model was constructed to train and cluster the relative resistance changes in response to difficult liquids, including acetone, DMF, ethyl alcohol and distilled water. The details of the artificial neural network model are provided in Fig. S30 and Table S3. The relative resistance change data associated with each liquid were randomly divided into a training set (80%), a testing set (10%) and a validation set (10%). Each sample used for training and testing undergoes preprocessing, which includes subtracting the mean and normalizing. By using this machine learning structure, the signal classification accuracy converged to 91.03% after 1600 batches training without overfitting (Fig. 6f-g). To further demonstrate the effectiveness and versatile sensing capabilities of this liquid sensor, the relative resistance response from mixed liquid (a volume ratio of 1/1) is investigated and enumerated to elucidate the volatilization characteristics of multiple liquids (Fig. 6h-k). For representative comparison, the test fluid contains highly volatile liquids and non-volatile liquid. Accordingly, Fig. 6h, i displays a stable trend over time due to the non-volatility of distilled water. With the introduction of highly volatile liquid, the relative resistance signals for acetone + DMF and acetone + ethyl alcohol mixtures decay within 71 and 112 s, respectively. The relative resistance change ratio (*h*_*1*_ = 2*h*_*2*_) in Fig. 6k exhibits a strong correlation with the volume ratio of acetone and ethyl alcohol, corresponding to the fully volatilization of acetone at time *t*_*2*_. The experimental results further provide an ideal sensing ability and the comprehensively quantified identification of the complex liquid systems in practical applications.

Conventional non-degradable polymer/mental-based sensing materials pose substantial challenges in terms of maintenance and environmental recycling in unmanned and distributed production environments, such as future unmanned factories where large-scale flexible sensing networks. In contrast, BC-based degradable fiber enables environmentally friendly decommissioning and replacement after long-term application, thereby preventing material accumulation and secondary pollution in complex industrial environments. This advantage is highly conductive to the development of green manufacturing and sustainable industrial IoTs. Therefore, the biodegradability of PKM_7_P_3_BC macrofibers as sensing units hold significant importance and enormous potential for the development of fully degradable textile-based sensor for unmanned factories and intelligent wearables. Bacterial cellulose can be biodegraded into glucose under the action of cellulase accompanied by the hydrolysis of *β*-1,4 glycoside bonds [58]. The biodegradability of PKM_7_P_3_BC macrofibers is evaluated by enzymatic degradation method. Although a shell layer exists between the liquid and the hydrophobic interface, partial solid–liquid contact sits are still present [59]. In addition, the entangled structure formed between the nanofibers and conductive fillers in the PKMPBC macrofiber results in only a limited fraction of nanofibers being exposed at the solid-liquid interface, allowing limited contact with cellulase and therefore causing a relatively slow initial degradation. As biodegradation progresses at these exposed sites, more nanofibers gradually become accessible to the cellulase solution, which in turn accelerates the overall degradation process. Real-time and SEM images of the degradation process of BC and PKM_7_P_3_BC macrofibers every 12 h are recorded in Figs. S31 and S32, it can be seen that PKM_7_P_3_BC macrofibers slowly break into several short fiber bundles within 60 h, and the internal BC nanofibers inside of macrofibers are gradually degraded. Then, K-MXene/PEDOT:PSS conductive aggregates begin to disintegrate in the cellulase solution until complete degradation within 132 h accompanied by black residues of K-MXene and PEDOT:PSS. The weight change of the macrofibers during degradation is also recorded (Fig. S33). The mass of PKM_7_P_3_BC macrofibers decreases by approximately 50% within 36 h and by 60% within 60 h, which shows the same trend as the optical and SEM images. The biodegradability study of BC and PKMPBC macrofibers confirms that PKMPBC macrofibers retains the intrinsic biodegradability of BC macrofibers, despite the presence of a shell coating.

## Conclusions

In this work, we report the judicious design and fabrication of highly conductive PKMPBC macrofiber electrode for applications in AIoT. The PKMPBC macrofibrous electrode is developed by integrating a homogeneous and highly conductive ink composed of KH570-modified MXene and PEDOT:PSS with BC macrofibers as the core and PDMS serves as the shell, where BC macrofibers provide mechanical strength, K-MXene/PEDOT:PSS ink acts as a conductive filler and mechanical enhancer and PDMS imparts durable hydrophobicity. The homogeneous conductive ink primarily originates from the strong π–π stacking interactions between PEDOT:PSS and K-MXene, as well as the structural transformation of PEDOT from a benzenoid configuration to a quinoid configuration. As a result, the K-MXene/PEDOT:PSS-integrated hydrophobic conductive macrofibers exhibit high electrical conductivity (10.05 S cm^−1^), superior mechanical strength (433.8 MPa) and a high Young’s modules (25.9 GPa). Furthermore, a self-powered PKT-TENG with excellent triboelectric response and output stability is engineered by integrating PKMPBC and PVDF-HFP nanofibers as triboelectric layers. The PKT-TENG delivers a maximum open-circuit voltage of 272.54 V, a short-circuit current of 14.56 μA and a peak power density of 86.29 mW m^−2^, successfully powering an electronic watch and commercial capacitors. Benefiting from the hydrophobicity, flexibility and self-cleaning capability inherited from PKMPBC macrofibers, the PKT-TENG maintains stable output even under harsh conditions such as mechanical bending and liquid intrusion. Additionally, a smart garment based on PKT-TENG is demonstrated for monitoring motion signals and is further extended into a motion and health monitoring system capable of recognizing motion posture and frequency. As a proof of concept, a PKMPBC-based liquid-recognition system is constructed for unmanned factory settings, where multiple liquid features of liquids (type, falling height and volume) are converted into relative resistance variations for signal recording, transmission and analysis, enabling robots to perform preliminary liquid classification. With these advantages, PKMPBC textiles, especially when integrated with wireless data transmission and intelligent signal processing, hold great promise for narrowing the gap between artificial electronic skin and human tactile perception, offering broad prospects in military defense, emergency rescue, intelligent manufacturing, and daily healthcare.

## Supplementary Information

Below is the link to the electronic supplementary material.

Supplementary file1 (DOCX 7831 KB)

Supplementary file2 (MP4 12191 KB)

Supplementary file3 (MP4 2533 KB)

Supplementary file4 (MP4 14185 KB)
