# Supplementary material for: Strong and Tough MXene-Induced Bacterial Cellulose Macrofibers for AIoT Textile Electronics
Source: Nanomicro Lett. 2026 Jan 7;18:198. doi: 10.1007/s40820-025-02046-y (PMC12779806; doi:10.1007/s40820-025-02046-y)
Supplement: Supplementary file 1 — Supplementary file1 (DOCX 7831 KB) [file 40820_2025_2046_MOESM1_ESM.docx]

Supporting Information for

**Strong and Tough MXene Induced Bacterial Cellulose Macrofibers for AIoT Textile Electronics**

Yi Hao^1, 2, 3^, Zixuan Zhang^2, 3^, Yajun Chen^4^, Song Wang^5^, Yingjia Tong^6^, Pengfei Lv^1, 7,^ *, Qufu Wei^1,^ * and Chengkuo Lee^2, 3,^ *

^1^ Key Laboratory of Special Protective Textiles, Ministry of Education, College of Textile Science and Engineering, Jiangnan University, Wuxi 214122, P. R. China

^2^ Department of Electrical and Computer Engineering, National University of Singapore, Singapore 117583, Singapore

^3^ Center for Intelligent Sensors and MEMS (CISM), National University of Singapore, Singapore 117583, Singapore

^4^ Suzhou Institute of Trade & Commerce, Suzhou 215009, P. R. China

^5^ State Key Laboratory for Mechanical Manufacturing Systems Engineering, Xi’an Jiaotong University, Xi’an 710049, P. R. China

^6^ School of Life Sciences and Health Engineering, Jiangnan University, Wuxi 214122, P. R. China

^7^ Laboratory of Flexible Electronics Technology, Tsinghua University, Beijing 10084, P. R. China

*****Corresponding authors. E-mail: [pengfeilv@jiangnan.edu.cn](mailto:pengfeilv@jiangnan.edu.cn) (Pengfei Lv); [qfwei@jiangnan.edu.cn](mailto:qfwei@jiangnan.edu.cn) (Qufu Wei); [elelc@nus.edu.sg](mailto:elelc@nus.edu.sg) (Chengkuo Lee)

**Supplementary Figures and Tables**


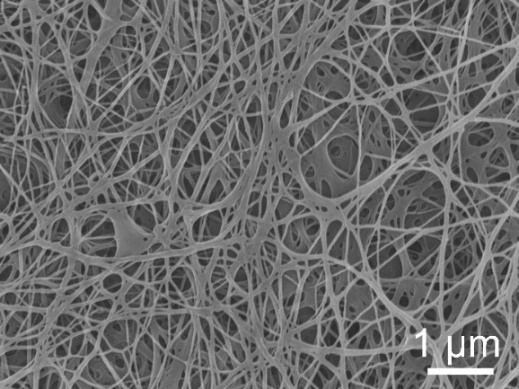


**Fig. S1** The surface morphology SEM image of BC micro-nanofiber membrane


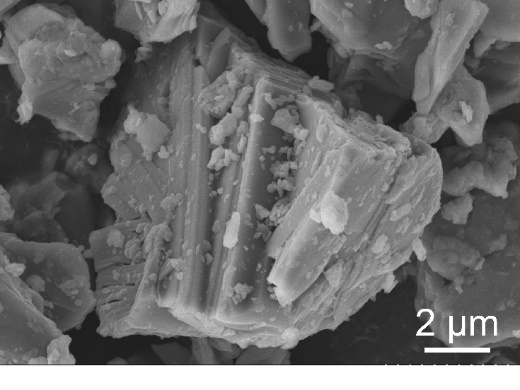


**Fig.** **S2** SEM image of bulk Ti_3_AlC_2_ MAX. A typical accordion-like structure can be clearly seen


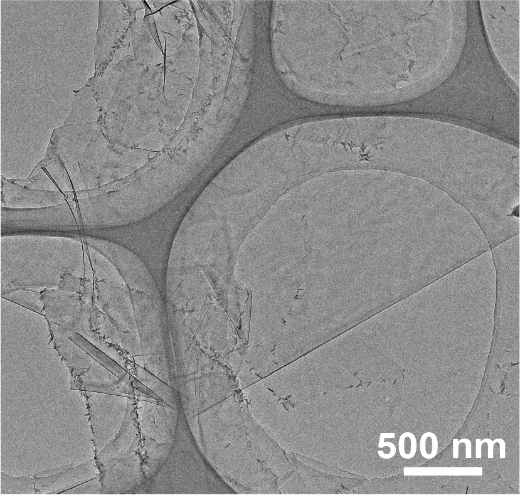


**Fig.** **S3** TEM image of exfoliated Ti_3_C_2_T_x_ MXene nanosheets


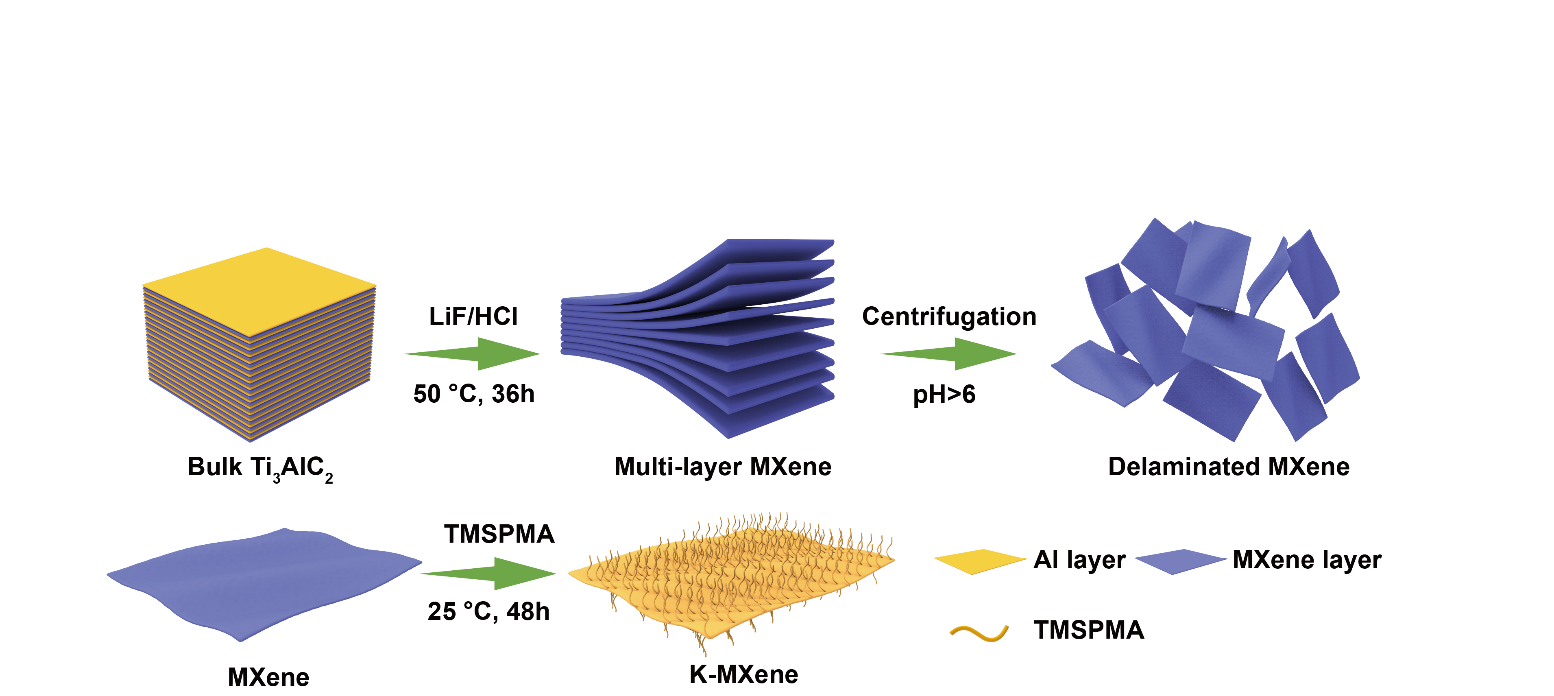


**Fig. S4** Schematic process of MXene nanosheets and surface-functionalized K-MXene single layers

Typically, 1.0 g Ti_3_AlC_2_ powder was slowly added to an etching solution consisting of 1.6 g of LiF and 20 mL of 9M HCl. After etching at 50 ℃ for 30 h, the mixture was washed with deionized water and repeatedly centrifuged at 3500 rpm for 10 min until the pH of the supernatant was approximately 6. Subsequently, anhydrous ethanol was added to the precipitate, and the mixture was ultrasonicated for 1 h to delaminate the Ti_3_C_2_T_x_ nanosheets. Finally, the delaminated single-layer Ti_3_C_2_T_x_ nanosheets were collected by centrifuging at 3500 rpm for 3 min several times, yielding a dark green dispersion of Ti_3_C_2_T_x_ MXene nanosheets. As a result, the final sediments were redispersed in DI water, resulting a 10 mg mL^-1^ concentration solution.


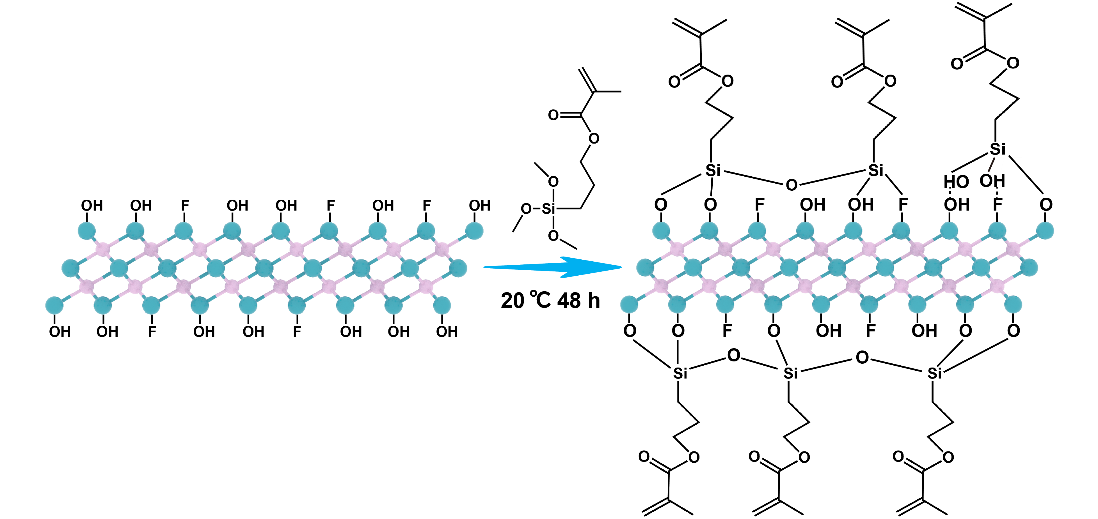


**Fig. S5** Schematic diagram of the surface functionalization of MXene nanosheets by the introduction of KH570


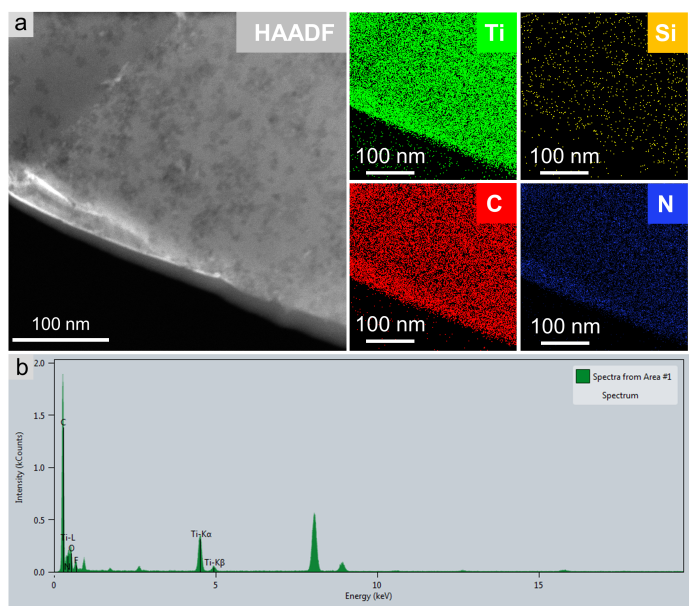


**Fig. S6** HAADF-STEM-EDS elemental maps of the K-MXene and the corresponding images of Ti, Si, C and N, respectively





**Fig. S7** XPS spectra of MXene and K-MXene films. X-ray photoelectron spectroscopy (XPS) reveals the appearance of new silicon peak in KH570, originating from the KH570 modifier


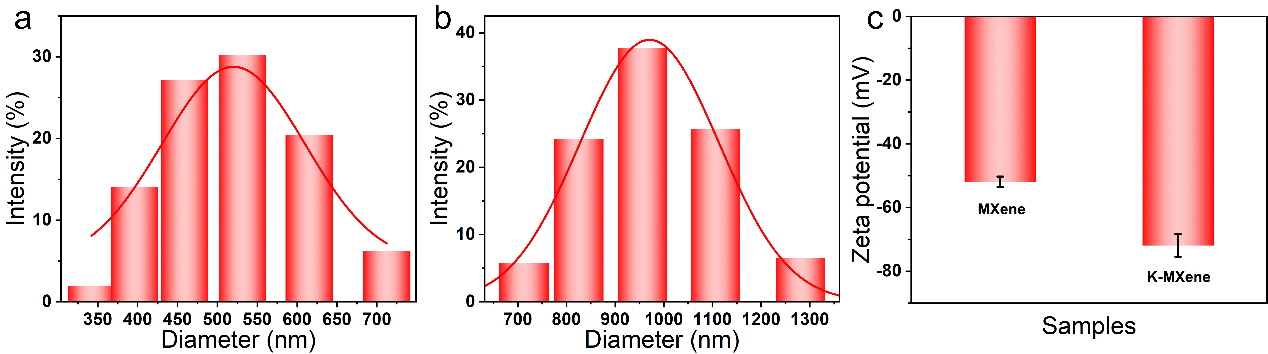


**Fig. S8** **a** Particle size distribution of MXene nanosheets and **b** K-MXene nanosheets dispersed in deionized water; **c** Zeta potential of MXene nanosheets and K-MXene nanosheets in deionized water


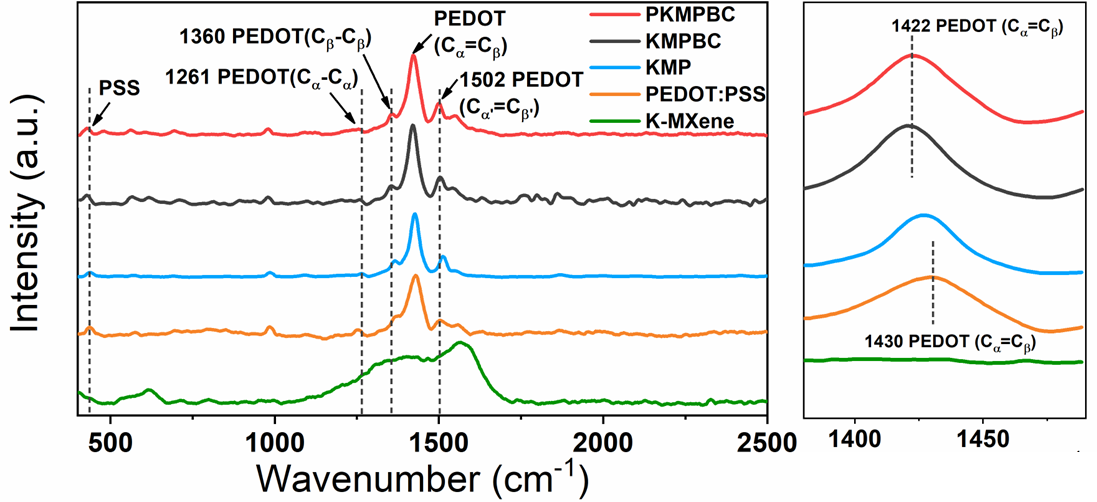


**Fig. S9** Raman spectra of K-MXene, PEDOT:PSS, KMP conductive ink, KMPBC and PKMPBC macrofibers


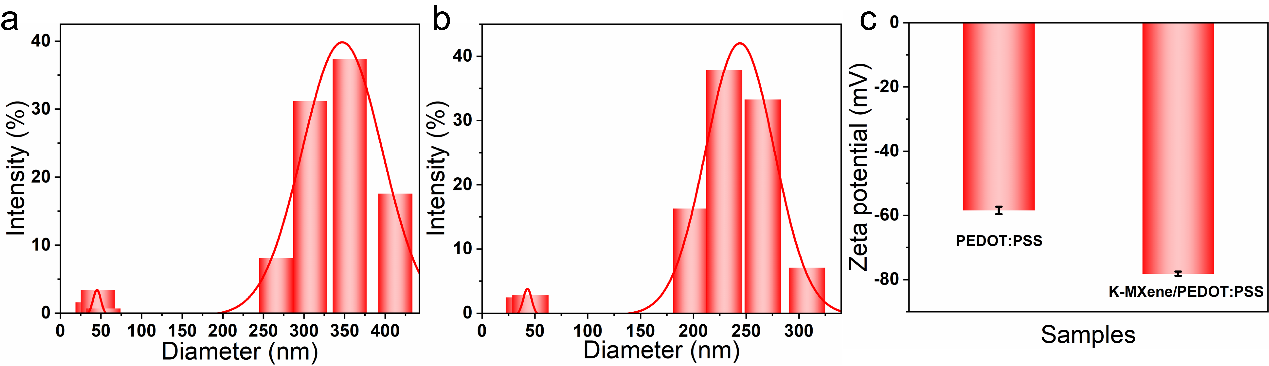


**Fig. S10 a** Particle size distribution of PEDOT:PSS dispersion and **b** K-MXene/PEDOT:PSS ink dispersed in deionized water; **c** Zeta potential of PEDOT:PSS and K-MXene/PEDOT:PSS ink dispersed in deionized water


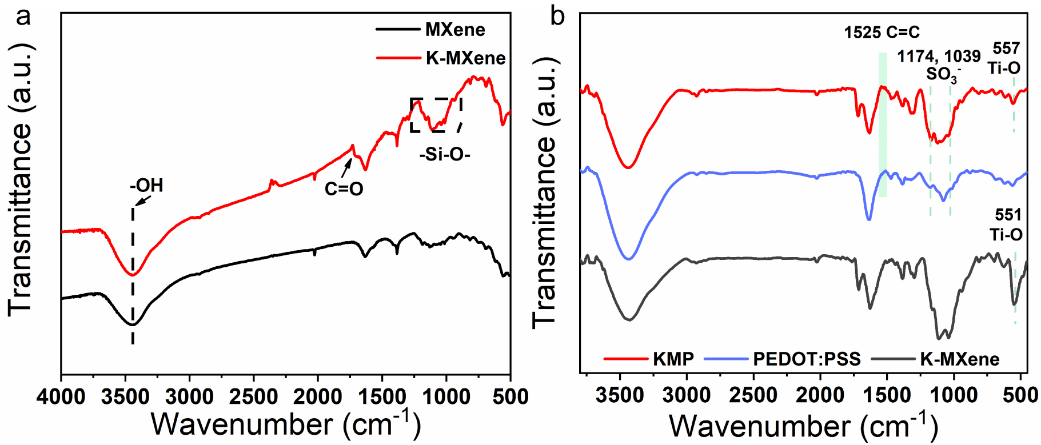


**Fig. S11** FTIR spectra of K-MXene, PEDOT:PSS, and KMP conductive ink. The KMP conductive ink clearly showed the C=C bond on the PSS benzene ring at 1525 cm⁻¹, and the SO_3_^⁻^ bond of the PSS benzene ring at 1174 cm⁻¹ and 1039 cm⁻¹, respectively. In addition, the Ti-O bonds representing the MXene nanosheets were also detected at 557 cm⁻¹





**Fig. S12** Thermogravimetric analysis (TGA) curves of MXene, BC macrofiber, KMBC macrofiber, KMPBC macrofiber and PKMPBC macrofiber

As shown in **Fig. S12**, the TGA curve of MXene nanosheet illustrates no obvious weight loss through entire temperature range with the residual weight maintaining a stable value of 89.5% at 800 °C. The weight of BC macrofibers reduced significantly at 290.6 °C, and the remaining weight is 19.8% at 800 °C. As a result, the thermal weight loss ratio of PKM_7_P_3_BC macrofibers is 46.8%.


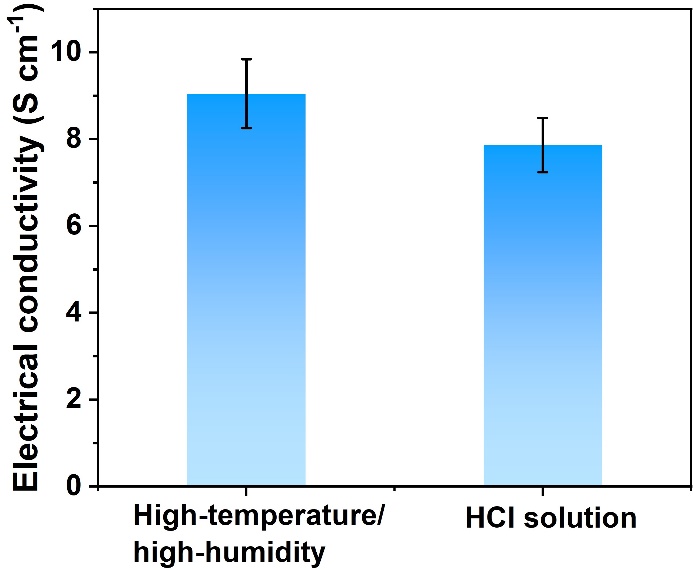


**Fig. S13** Electrical conductivity of PKMPBC in high-temperature/high-humidity (40±2 °C, 90%±3%) and 10% (w/w) HCl solution, respectively


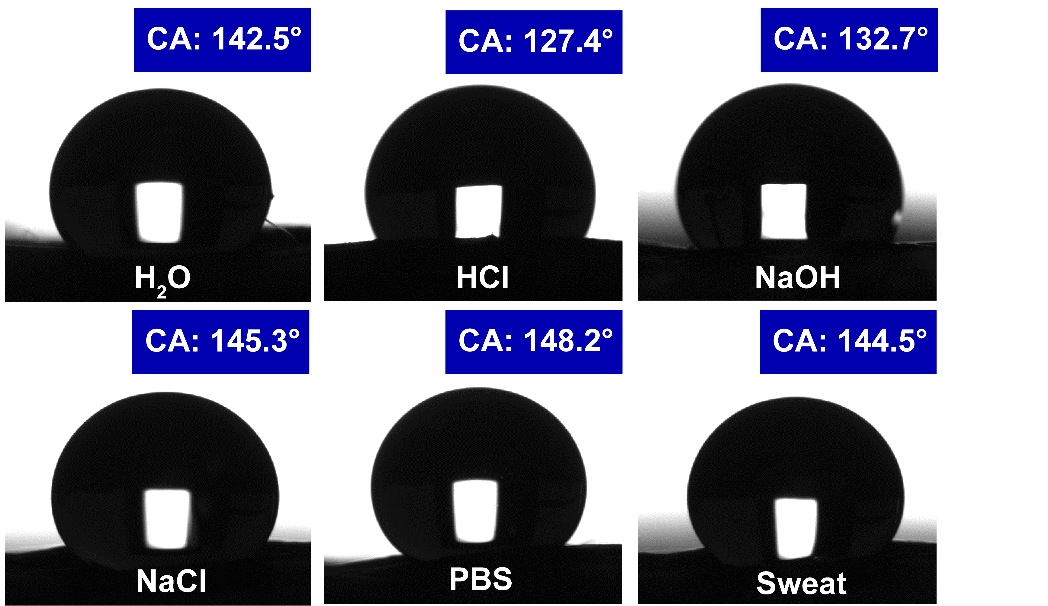


**Fig. S14** Contact angle images of PKMPBC fabric with water, HCl solution, NaOH solution, NaCl solution, PBS buffer solution and sweat, respectively


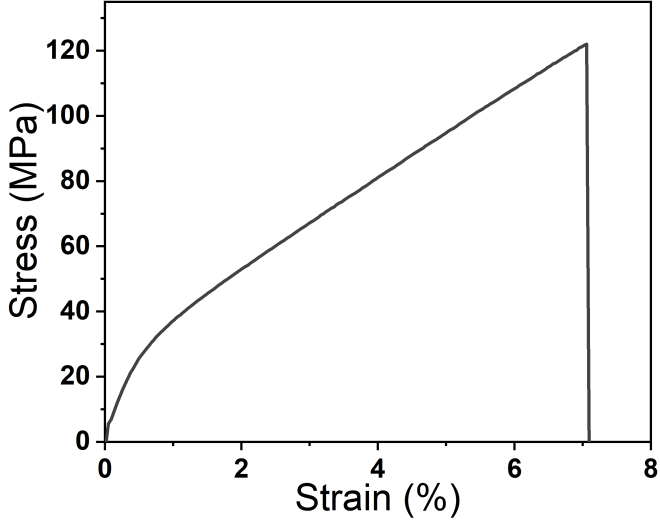


**Fig. S15** Stress-strain curve of undrawn BC macrofibers


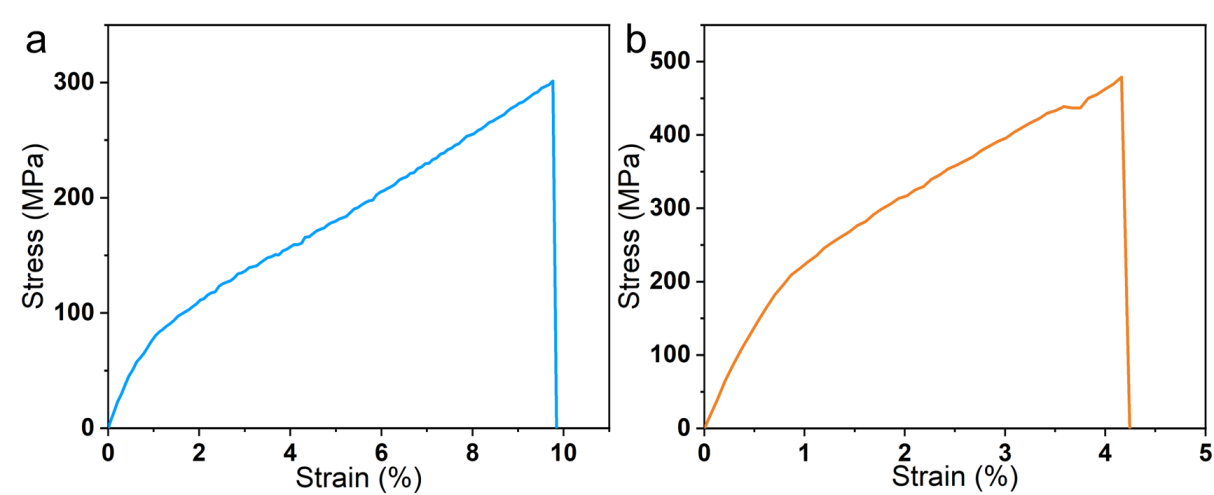


**Fig. S16** Stress-strain curve of PKMPBC macrofibers under (a) high temperature and high humidity (40±2 °C, 90%±3%) and (b) 10% (w/w) HCl solution

**
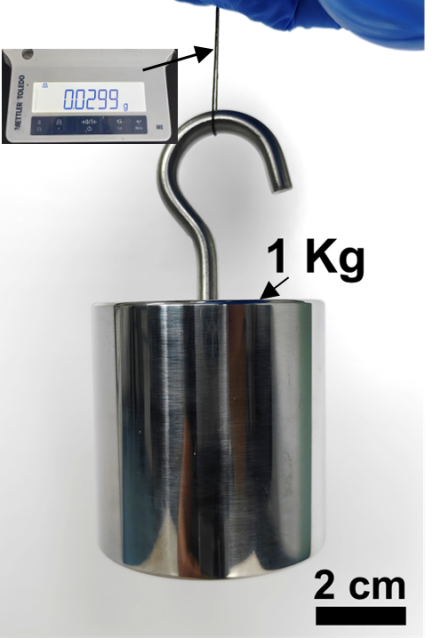
**

**Fig. S17** A PKM_7_P_3_BC macrofiber of 29.9 mg can lift up a weight of 1 kg which is more than 30000 times heavier than the macrofiber

**
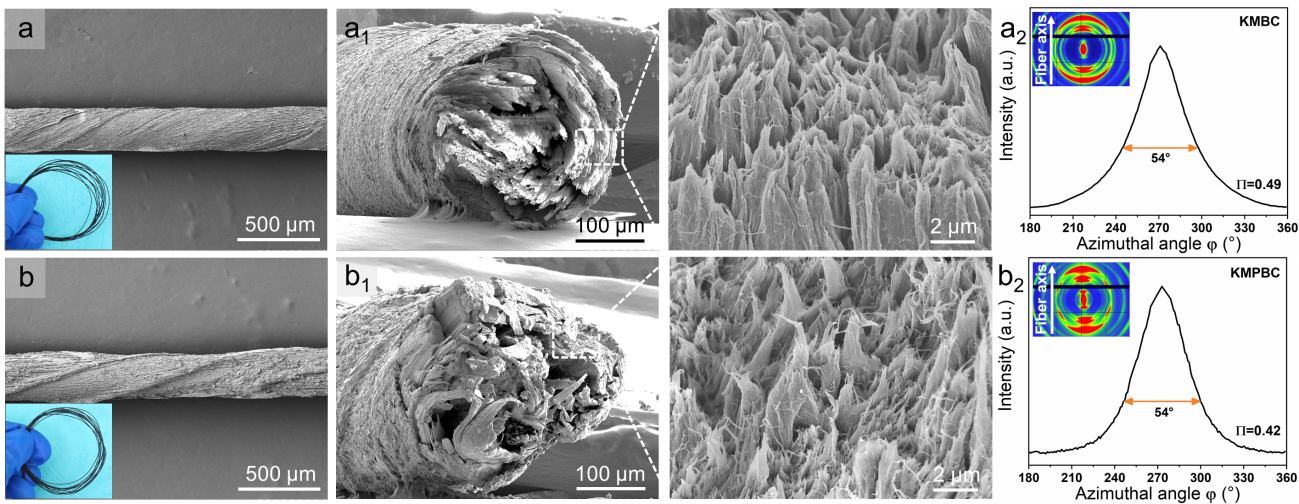
**

**Fig. S18 a, b** SEM images, **a_1_, b_1_** Cross-sectional and enlarged SEM images of KMBC and KMPBC macrofibers, and **a_2_, b_2_** 2D WAXS images with corresponding *fwhm* curves


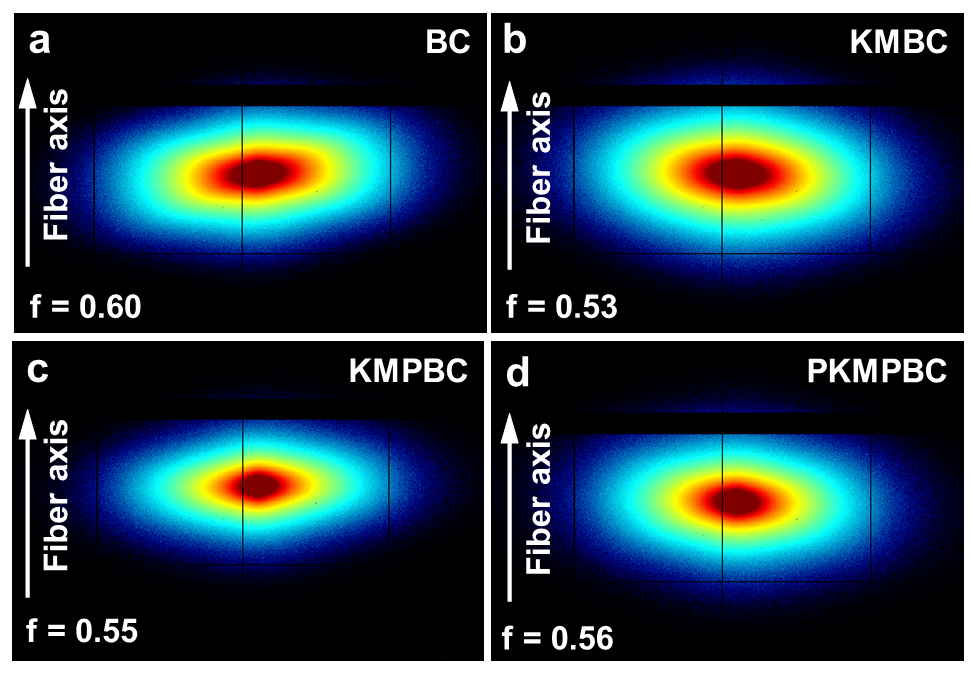


**Fig. S19** The 2D SAXS images of **a** BC, **b** KMBC **c** KMPBC and **d** PKMPBC fibers


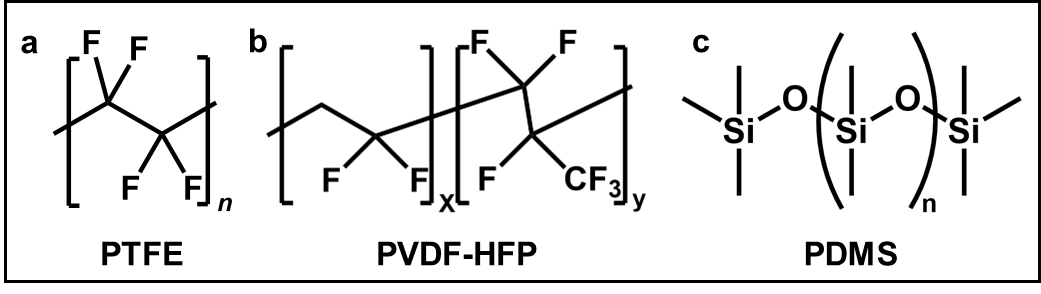


**Fig. S20** The detailed molecular structure of **a** PTFE, **b** PVDF-HFP and **c** PDMS, respectively


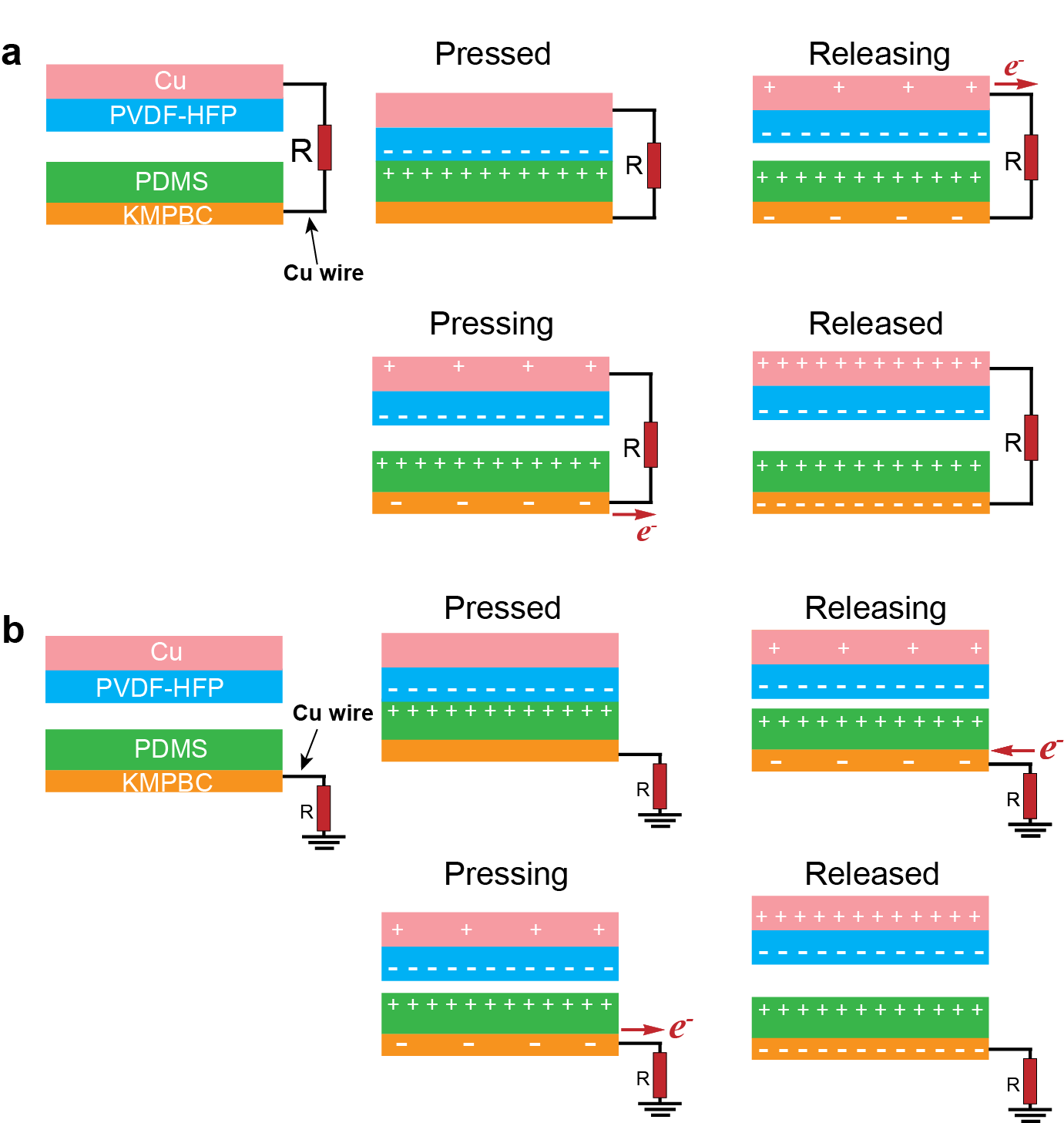


**Fig. S21** Working principle of PKT-TENG in **a** double-electrode mode, **b** single-electrode mode


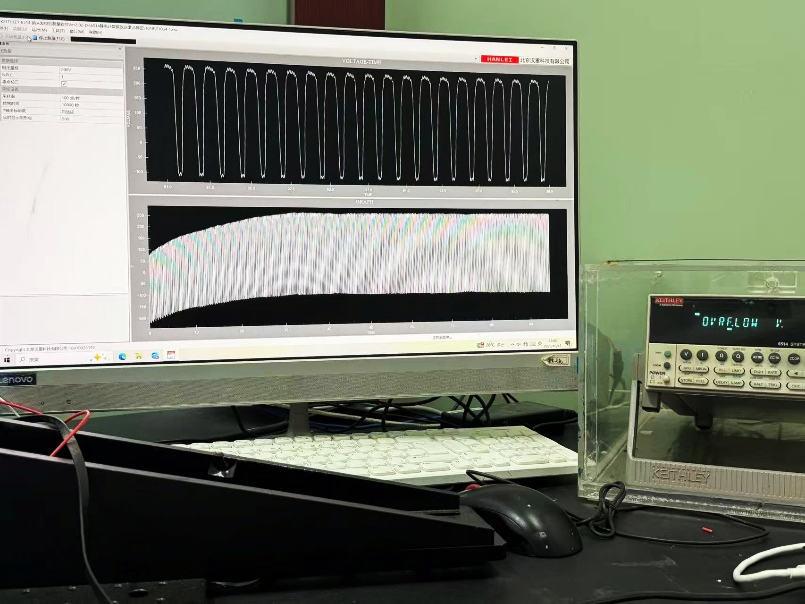


**Fig. S22** The output voltage testing photograph of PKT-TENG at a working frequency of 4 Hz





**Fig. S23** Relationship between the output voltage and current of PKMPBC fabric-based TENG when connected to different external resistances ranging from 0.1 MΩ to 1000 MΩ


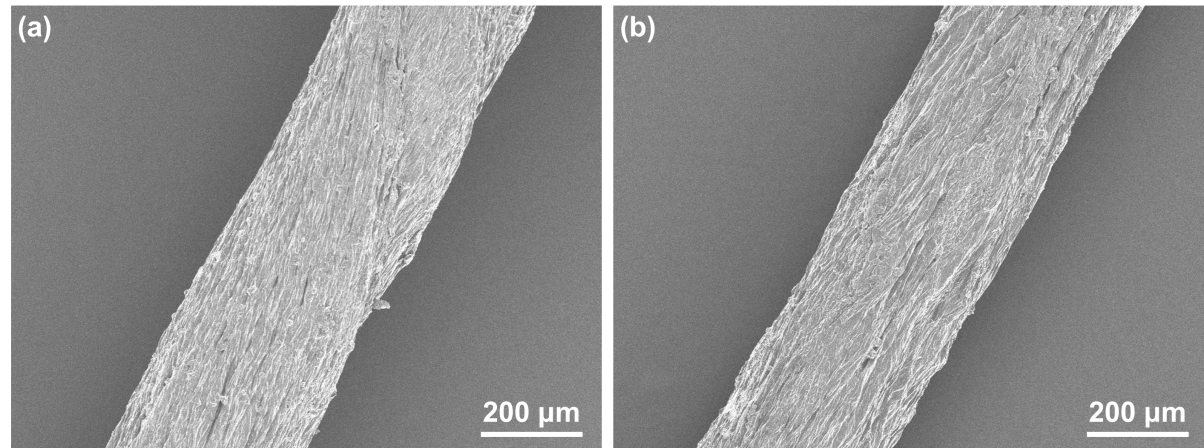


**Fig. S24** The variation in the SEM surface morphology of the PKMPBC macrofiber (a) before and (b) after 2700 contact-separation cycles





**Fig. S25** Output voltage of PF-TENG before and after water injection on the friction surface





**Fig. S26** Charging curves of PKM_7_P_3_BC textile-based TENG for commercial capacitors at different operating frequencies (0.5 Hz-3.0Hz)


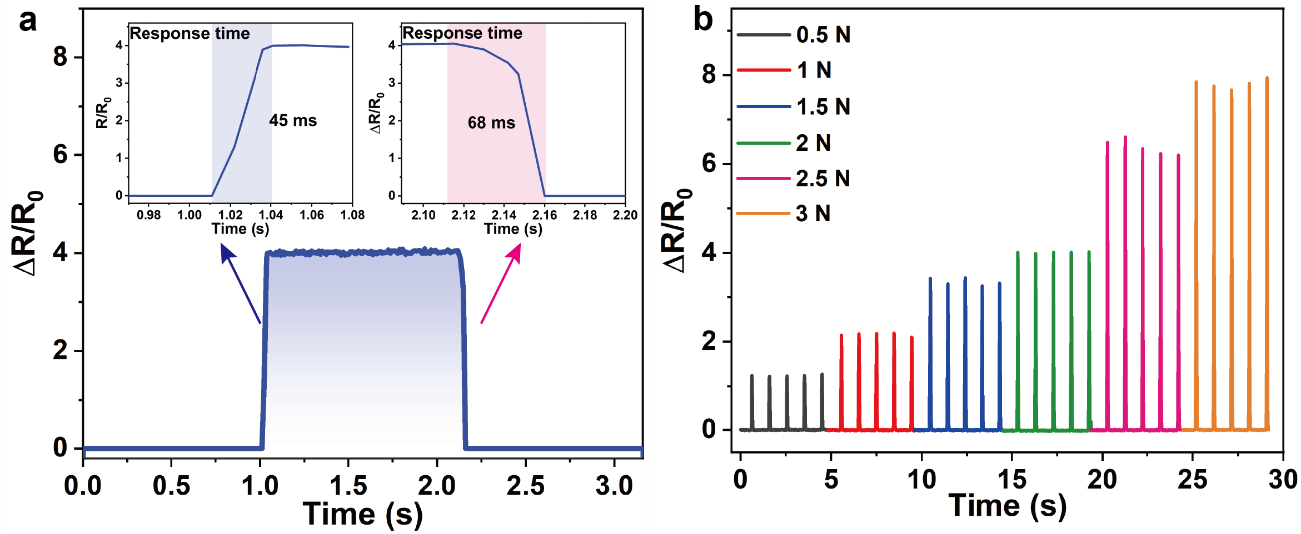


**Fig. S27 a** The response and recovery time of the PKMPBC textile-based sensor under finger pressure. **b** Cyclic relative resistant change of the PKMPBC textile-based sensor at diverse loads from 0.5 N to 3 N

The operational performance of the PKMPBC textile-based sensor was evaluated under ambient conditions (20±3°C, 40%±2%). When a forefinger touched the surface of PKMPBC textile-based sensor, an ultrafast response in resistance variation was observed within 45 ms. Subsequently, upon removal of the finger, the resistance returned to its initial level within 68 ms without any baseline drift (Fig. S27a). Fig. 27b exhibits the relative resistance changes of the PKMPBC textile-based sensor under cyclic loads in the range of 0.5-3 N. The signal peak of the relative resistant changes can clearly distinguish pressures of different amplitudes and present the repeatability of the relative resistant changes at the identical load. These illustrate that the PKMPBC textile-based sensor possesses reliable monitoring and a stable sensing response for full-range monitoring induced by the environment stimuli.


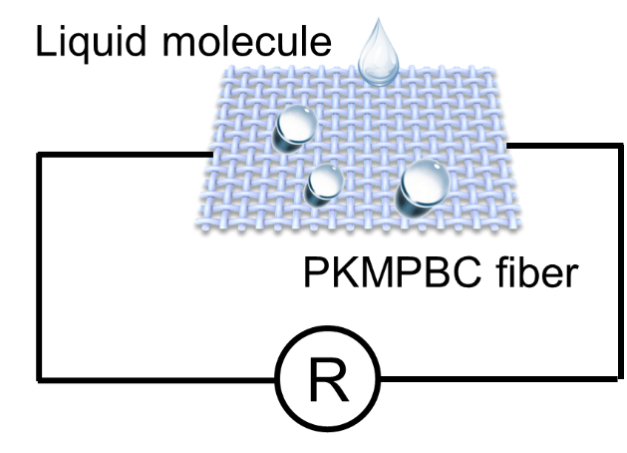


**Fig. S28** Schematic diagram of liquid molecule recognition sensing system circuit (3 cm×2.5 cm)


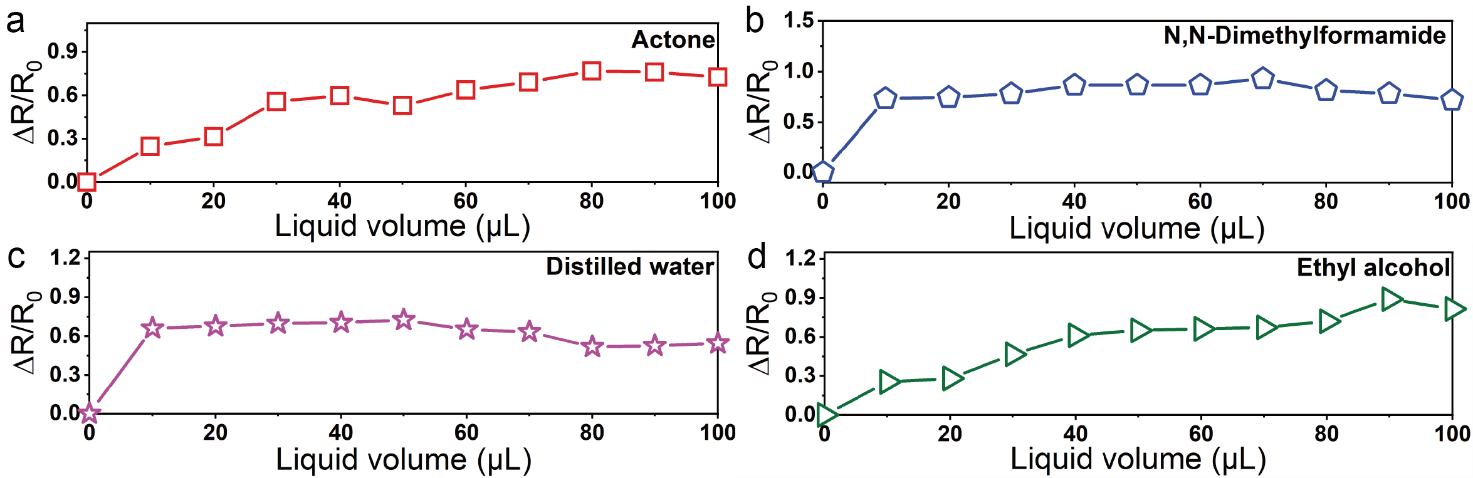


**Fig. S29** Resistance changes in the recognition system at different liquid volume (0-100 μL)


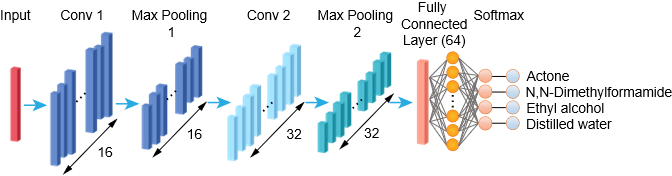


**Fig. S30** Snapshots of the real classification system and process


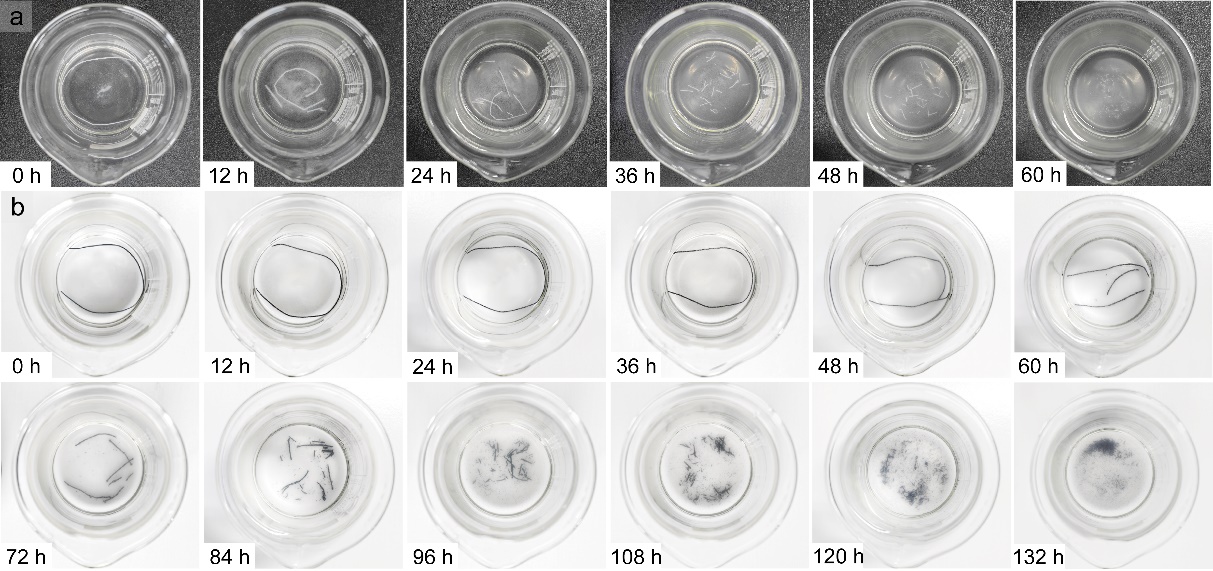


**Fig. S31** Optical photographs of (**a**) BC and (**b**) PKPMBC macrofibers in the degradation experiment with the cellulase solution

**
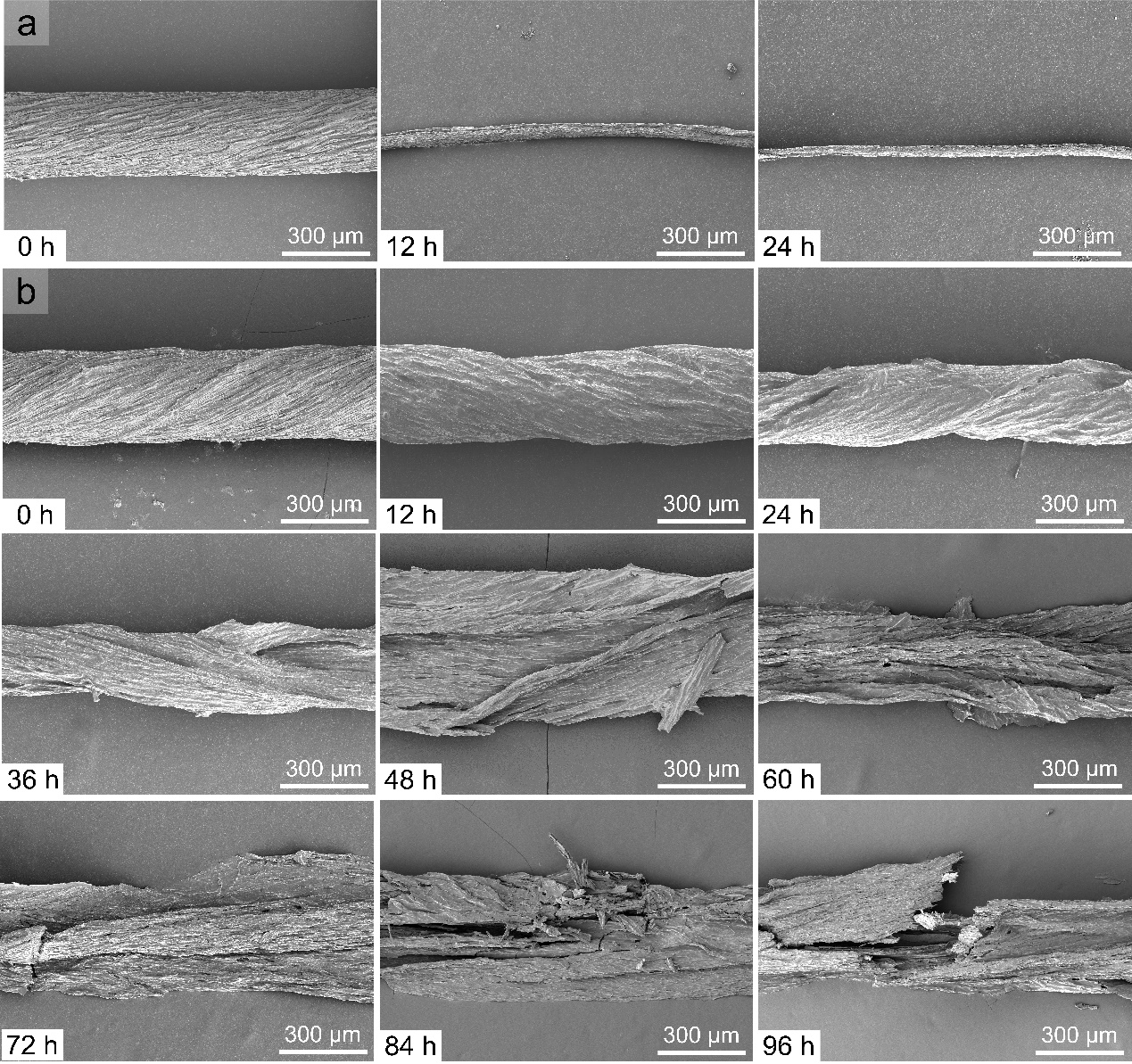
**

**Fig. S32** SEM images of (**a**) BC and (**b**) PKMPBC macrofibers in the degradation experiment with cellulase solution


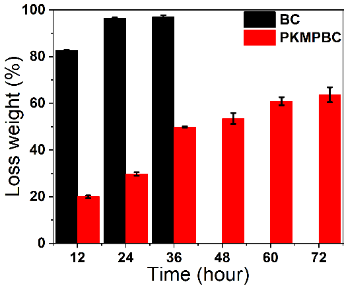


**Fig. S33** The weight loss of the BC and PKMPBC macrofibers during degradation

The real-time images of the degradation process of BC and PKM_7_P_3_BC macrofibers every 12 hours are recorded in **Fig. S31**, it can be seen that the BC and PKM_7_P_3_BC macrofibers gradually degrade in cellulase solution. The SEM images of the degradation process of BC macrofibers in 24 h (**Fig. S32**) indicates that the diameter of BC macrofiber gradually decreases. During this process, the macrofibers simultaneously fragmenting until completely degradation. It can be clearly observed that the degradation process of PKM_7_P_3_BC macrofiber is slower than that of BC macrofiber because of the K-MXene/PEDOT:PSS ink and PDMS hydrophobic shell coating. Specifically, the PKM_7_P_3_BC macrofiber slowly break into several short fiber bundles within 60 h, and the internal BC nanofibers inside of macrofibers are gradually degraded. Then, K-MXene/PEDOT:PSS conductive aggregates begin to disintegrate in the cellulase solution until complete degradation within 132 h accompanied by black residues of K-MXene and PEDOT:PSS. Furthermore, the weight change of the macrofibers during degradation is also recorded, as shown in **Fig. S33**. During the degradation process, the mass of BC macrofibers decreases by 82% within 12 h and by 96% within 24 h. The degradation rate of BC macrofibers slows down as it approached complete degradation from 12 h to 24 h, whereas the PKM_7_P_3_BC macrofibers maintains a stable degradation rate. The mass of PKM_7_P_3_BC macrofibers decreases by approximately 50% within 36 h and by 60% within 60 h. The mass changes of BC and PKM_7_P_3_BC macrofibers in weight loss curve shows the same trend as the optical and SEM images.

**Table S1** The comparison of different properties among different fibers

| **Fiber** | **Mechanical strength (MPa)** | **Conductivity (S cm^-1^)** | **Strain (%)** | **Young's modulus (GPa)** | **Output**  **voltage** | **Applications** | **Refs.** |
| --- | --- | --- | --- | --- | --- | --- | --- |
| MWCNTs/ CSF | 0.4 | 1 | 1378 | 0.49 GPa | / | Strain sensor | [S1] |
| MXene/CNT (95wt% MXene) | 38.4 | 2.7 | 4.7 | / | / | Piezoresistive sensor | [S2] |
| ANFs/MXene (2:1) | 20 | 0.171 | 2.8 | 0.395 GPa | / | Resistance sensor | [S3] |
| ANFs/MXene (1:1) | 20 | 0.117 | 6.8 | 0.75 GPa | / | Resistance sensor | [S3] |
| ANFs/MXene (1:2) | 13 | 0.05 | 7.5 | 1.74 GPa | / | Resistance sensor | [S3] |
| MXene | 8 | 10 | 0.46 | 20.30 GPa | 160 mV | Strain sensor | [S4] |
| MXene/TPU | 3.76 | 4.32 | 675 | 0.76 GPa | 20.1 V | TENG & biomechanical sensor | [S5] |
| MWCNTs/cellulose | 449 | 5.32 | 3 |  | 170 V | TENG | [S6] |
| MWCNTs/aramid fiber | 47.2 | 82 | 9.6 | / | / | Electric heater | [S7] |
| MXene/bacterial cellulose paper | 109.6 | 50.2 | 11.2 | 2.4 GPa | / | Electromagnetic shielding | [S8] |
| d-Ti_3_C_2_T_X_/CNFs | 135.4 | 7.39 | 16.7 | 3.8 GPa | / | Electromagnetic shielding | [S9] |
| MXene/rGO (88 wt% MXene) | 132.5 | 72.3 | 2.9 | / | / | Supercapacitors | [S10] |
| MXene/GO/cellulose fiber | 162.5 | 368.2 | 6.7 | 9.0 GPa | / | Electromagnetic shielding | [S11] |
| MXene/PEDOT:PSS | 58.7 | 1489 | 2 | 7.5 GPa | / | Supercapacitors | [S12] |
| **PKMPBC macrofiber** | **434** | **10.1** | **4.1** | **25.9 GPa** | **272.5 V** | **TENG & biomechanical sensor** | **This work** |

**Table S2** The combination properties comparison with other similar materials

| **Fiber** | **Response/**  **recovery time** | **Filler contents** | **Conductivity** | **Permeability** | **Stability** | **Refs.** |
| --- | --- | --- | --- | --- | --- | --- |
| Kevlar/  MXene | 90 ms/110 ms | 1 wt% | 0.17 S m^-1^ | / | / | [S13] |
| CNT/MXene/TPU | 89 ms | 4 wt% (CNT); 1 wt% (MXene) | 13.1 S cm^-1^ | / | 1000 | [S14] |
| CB/MXene/  plant fiber@PDMS | 40 ms/100 ms | 30 wt% | ≈ 2kΩ | / | 5000 | [S15] |
| MXene/GO-AgNWs | 69 ms/71 ms | MXene: 10 mg mL^-1^ |  | 800±50 mm/s | 1000 | [S16] |
| MXene  /PVA | / | MXene: 40 wt% | 0.28 S cm^-1^ | / | / | [S17] |
| **PKMPBC macrofiber** | **45 ms/68 ms** | **MXene: 20 mg mL^-1^** | **10.05 S cm^-1^** | **/** | **2700** | **This work** |

**Table S3** The parameters for constructing Convolution Netural Network (CNN)

| **Layer Type** | **Parameters** | **Output Shape** |
| --- | --- | --- |
| Input | Length = 4000, Channels = 1 | [4000, 1] |
| 1D Convolution + BN + ReLU | Kernel Size = 5, Filters = 16 | [4000, 16] |
| MaxPooling1D | Pool Size = 2, Stride = 2 | [2000, 16] |
| 1D Convolution + BN + ReLU | Kernel Size = 5, Filters = 32 | [2000, 32] |
| MaxPooling1D | Pool Size = 2, Stride = 2 | [1000, 32] |
| Global Average Pooling 1D | - | - |
| Fully Connected (Dense) | Units = 64 | [64] |
| ReLU Activation | - | - |
| Fully Connected (Dense) | Units = 4 (number of classes) | [4] |
| Softmax | - | [4] |
| Classification Layer | Cross-entropy loss | [4] |

**Supplementary Movies**

**Movie S1** The flexibility of PKT-TENG

**Movie S2** Output performance of PKF-TENG under pouring liquids

**Movie S3** Detecting motion signals after pouring water

**Supplementary References**

1. Z. Tang, S. Jia, F. Wang, C. Bian, Y. Chen et al., Highly stretchable core-sheath fibers *via* wet-spinning for wearable strain sensors. ACS Appl. Mater. Interfaces **10**(7), 6624–6635 (2018). <https://doi.org/10.1021/acsami.7b18677>
2. Y. Ma, N. Liu, L. Li, X. Hu, Z. Zou et al., A highly flexible and sensitive piezoresistive sensor based on MXene with greatly changed interlayer distances. Nat. Commun. **8**(1), 1207 (2017). <https://doi.org/10.1038/s41467-017-01136-9>
3. B. Cheng, P. Wu, Scalable fabrication of kevlar/Ti_3_C_2_T*_x_* MXene intelligent wearable fabrics with multiple sensory capabilities. ACS Nano **15**(5), 8676–8685 (2021). <https://doi.org/10.1021/acsnano.1c00749>
4. S. Seyedin, S. Uzun, A. Levitt, B. Anasori, G. Dion et al., MXene composite and coaxial fibers with high stretchability and conductivity for wearable strain sensing textiles. Adv. Funct. Mater. **30**(12), 1910504 (2020). <https://doi.org/10.1002/adfm.201910504>
5. Y. Hao, Y. Zhang, A. Mensah, S. Liao, P. Lv et al., Scalable, ultra-high stretchable and conductive fiber triboelectric nanogenerator for biomechanical sensing. Nano Energy **109**, 108291 (2023). <https://doi.org/10.1016/j.nanoen.2023.108291>
6. S. Hu, J. Han, Z. Shi, K. Chen, N. Xu et al., Biodegradable, super-strong, and conductive cellulose macrofibers for fabric-based triboelectric nanogenerator. Nanomicro Lett. **14**(1), 115 (2022). <https://doi.org/10.1007/s40820-022-00858-w>
7. B. Yang, X. Ding, M. Zhang, L. Wang, Scalable electric heating paper based on CNT/Aramid fiber with superior mechanical and electric heating properties. Compos. Part B Eng. **224**, 109242 (2021). <https://doi.org/10.1016/j.compositesb.2021.109242>
8. H. Liu, Z. Cui, L. Luo, Q. Liao, R. Xiong et al., Facile fabrication of flexible and ultrathin self-assembled Ti_3_C_2_T/bacterial cellulose composite films with multifunctional electromagnetic shielding and photothermal conversion performances. Chem. Eng. J. **454**, 140288 (2023). <https://doi.org/10.1016/j.cej.2022.140288>
9. W.-T. Cao, F.-F. Chen, Y.-J. Zhu, Y.-G. Zhang, Y.-Y. Jiang et al., Binary strengthening and toughening of MXene/cellulose nanofiber composite paper with nacre-inspired structure and superior electromagnetic interference shielding properties. ACS Nano **12**(5), 4583–4593 (2018). <https://doi.org/10.1021/acsnano.8b00997>
10. S. Seyedin, E.R.S. Yanza, J. Razal, Knittable energy storing fiber with high volumetric performance made from predominantly MXene nanosheets. J. Mater. Chem. A **5**(46), 24076–24082 (2017). <https://doi.org/10.1039/c7ta08355f>
11. L.-X. Liu, W. Chen, H.-B. Zhang, Y. Zhang, P. Tang et al., Tough and electrically conductive Ti_3_C_2_T*_x_* MXene–based core–shell fibers for high–performance electromagnetic interference shielding and heating application. Chem. Eng. J. **430**, 133074 (2022). <https://doi.org/10.1016/j.cej.2021.133074>
12. J. Zhang, S. Seyedin, S. Qin, Z. Wang, S. Moradi et al., Highly conductive Ti_3_C_2_T*_x_* MXene hybrid fibers for flexible and elastic fiber-shaped supercapacitors. Small **15**(8), 1804732 (2019). <https://doi.org/10.1002/smll.201804732>
13. B. Cheng, P. Wu, Scalable fabrication of kevlar/Ti_3_C_2_T*_x_* MXene intelligent wearable fabrics with multiple sensory capabilities. ACS Nano **15**(5), 8676–8685 (2021). <https://doi.org/10.1021/acsnano.1c00749>
14. L. Lan, C. Jiang, Y. Yao, J. Ping, Y. Ying, A stretchable and conductive fiber for multifunctional sensing and energy harvesting. Nano Energy **84**, 105954 (2021). <https://doi.org/10.1016/j.nanoen.2021.105954>
15. T. Chen, Z. Liu, G. Zhao, Z. Qin, P. Zheng et al., Piezoresistive sensor containing lamellar MXene-plant fiber sponge obtained with aqueous MXene ink. ACS Appl. Mater. Interfaces **14**(45), 51361–51372 (2022). <https://doi.org/10.1021/acsami.2c15922>
16. Z.M. Abay, Y. Wei, Z. Tang, Y. Liu, K. Chen et al., Versatile MXene/(GO-AgNWs) electronic textile enabled by mixed-scale assembly strategy. Nano Energy **139**, 110963 (2025). <https://doi.org/10.1016/j.nanoen.2025.110963>
17. M. Wei, N. Wu, B. Li, J. Liu, F. Pan et al., From MXene to multimodal-responsive smart, durable electromagnetic interference shielding textiles. Adv. Funct. Mater. **35**(28), 2424312 (2025). <https://doi.org/10.1002/adfm.202424312>
